# ILR-Net: Low-light image enhancement network based on the combination of iterative learning mechanism and Retinex theory

**DOI:** 10.1371/journal.pone.0314541

**Published:** 2025-02-13

**Authors:** Mohan Yin, Jianbai Yang

**Affiliations:** School of Computer Science and Information Engineering, Harbin Normal University, Harbin, Heilongjiang, China; Dalian Maritime University, CHINA

## Abstract

Images captured in nighttime or low-light environments are often affected by external factors such as noise and lighting. Aiming at the existing image enhancement algorithms tend to overly focus on increasing brightness, while neglecting the enhancement of color and detailed features. This paper proposes a low-light image enhancement network based on a combination of iterative learning mechanisms and Retinex theory (defined as ILR-Net) to enhance both detail and color features simultaneously. Specifically, the network continuously learns local and global features of low-light images across different dimensions and receptive fields to achieve a clear and convergent illumination estimation. Meanwhile, the denoising process is applied to the reflection component after Retinex decomposition to enhance the image’s rich color features. Finally, the enhanced image is obtained by concatenating the features along the channel dimension. In the adaptive learning sub-network, a dilated convolution module, U-Net feature extraction module, and adaptive iterative learning module are designed. These modules respectively expand the network’s receptive field to capture multi-dimensional features, extract the overall and edge details of the image, and adaptively enhance features at different stages of convergence. The Retinex decomposition sub-network focuses on denoising the reflection component before and after decomposition to obtain a low-noise, clear reflection component. Additionally, an efficient feature extraction module—global feature attention is designed to address the problem of feature loss. Experiments were conducted on six common datasets and in real-world environments. The proposed method achieved PSNR and SSIM values of 23.7624dB and 0.8653 on the LOL dataset, and 26.8252dB and 0.7784 on the LOLv2-Real dataset, demonstrating significant advantages over other algorithms.

## Introduction

Currently, computer vision tasks have made significant progress in the areas of target detection, image classification and image segmentation. However, these are built on the basis of well-lit daytime scenes, and images acquired or captured under conditions such as low light and backlighting usually face challenges such as low brightness, loss of details, and color shifts, which seriously affect the effectiveness of various vision tasks. Therefore, enhancement of images captured in low-light environments is of great significance and practical value.

**Fig 1:**
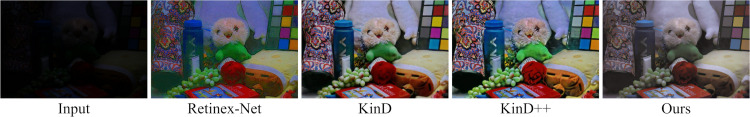
The image obtained by Retinex-Net has well-preserved color details, but there is noise; the image obtained by KinD and KinD++ has good denoising effect, but the details are blurred.

Over decades of progress within low-light image enhancement (LLIE) [1–3]. Numerous methodologies have emerged, encompassing techniques such as Histogram equalization(HE) [4], Retinex-based methods [5–8], deep learning approaches, among others. Among these, the Retinex theory [9–13] is commonly utilized to mimic human visual perception of objects and further decompose the image into reflection and illumination components. Therefore, the mathematical representation of image I can be expressed as:

I=R∘L (1)

where *L* represent the illumination component containing variations in image brightness and distribution of light intensity, and *R* represents the reflection component containing rich image details and color characteristics, and ∘ denotes element-wise multiplication. Although early Retinex-based algorithms can enhance image brightness, the image visual effect is poor, prone to a large amount of noise, and computationally complex. For this reason, scholars have developed an end-to-end LLIE enhancement network by combining deep learning with Retinex model and used the network to estimate and enhance the illumination and reflection maps respectively. However, it is difficult to simultaneously denoise and retain detailed information while maintaining the enhancement effect. This is shown in Fig 1:

In this paper, we propose ILR-Net, a low-light image enhancement network based on the combination of iterative learning mechanism and Retinex theory. The network consists of two sub-networks: adaptive learning and Retinex decomposition. In the adaptive learning sub-network, the original image undergoes initial feature extraction through a multi-branch dilated convolution module. The extracted features are then processed by feature enhancement units and a U-Net feature learning module for deeper-level learning. These outputs are subsequently passed into feature fusion units for information integration, resulting in a clear and converged image. Throughout this process, weight sharing is applied. In the Retinex decomposition sub-network, unlike traditional Retinex approaches that enhance the illumination component, we focus on directly denoising the reflection component, which contains rich detail information. The low-light image is first processed by a layer-by-layer denoising decomposition module, which integrates Coordinate Attention (CA) [14], Squeeze-and-Excitation Attention (SE) [15], and residual layers before decomposition. This step yields a reflectance map with detailed information. The reflectance map is then further processed through the reflectance component denoising module for feature extraction and denoising, guiding the fusion of the final enhanced image.

The contributions of this paper are as follows:

- This paper presents a novel LLIE network. The network contains adaptive learning sub-network and Retinex decomposition sub-network. Extensive experiments show that our method outperforms other state-of-the-art LLIE methods and exhibits good subjective visualization.
- The Global Feature Attention (GFA) is designed in the U-Net feature learning module inspired by the Convolutional Block Attention Module (CBAM) [16] to improve the extraction of image detail information and retain more feature information. An adaptive iterative learning module with weight sharing is designed, to realize the fusion convergence of the results at each stage, and to obtain a clearer image by learning through several iterations.
- Propose a layer-by-layer denoising decomposition subnetwork. The denoising operation is performed before image decomposition to obtain better decomposition results.

The subsequent content of this paper is outlined as follows: Section “Related Work” provides a categorization of pertinent methodologies for LLIE. Section “Proposed method” introduces the ILR-Net framework, detailing each module and the loss function. Section “Experimental results and analysis” presents the experimental results and analyses. Section “Conclusions” makes the conclusion section.

## Related work

Currently, there has been significant development in the field of LLIE, which can be broadly categorized into traditional low-light image enhancement and deep learning-based low-light image enhancement.

### Traditional methods

Faced with the challenges of computer vision tasks in low light environments, early scholars began to utilize traditional LLIE methods for image enhancement. These include histogram equalization-based enhancement and Retinex theory-based enhancement. Histogram Equalization (HE) [4] enhances the contrast of an image by redistributing the image pixel values to make the histogram of the image more homogeneous, thereby achieving better visibility. Examples include global histogram equalization, adaptive histogram equalization, and so on [17–19]. This method is fast and effective and does not require additional parameters, but it is accompanied by problems such as loss of details in local areas, poor enhancement, and intensity enhancement of noise in the image.

The Retinex theory [10–13] views an image as consisting of two components, the illumination component and the reflection component, and argues that the brightness and color of an image depend mainly on the reflective properties of the object, rather than on the lighting conditions alone. The illumination component is enhanced to obtain a corresponding normal light image. Variants of this theory include Single Scale Retinex (SSR) [5, 6], Multi Scale Retinex (MSR) [7], and Color Recovery based Retinex Algorithm (MSRCR) [8]. Although the above methods provide a good improvement in image enhancement, color restoration and detail preservation, the algorithm is slow and cannot be applied to some real-time scenes.

Therefore, in most cases, traditional enhancement methods rely too much on manually designed a priori or statistical models to a large extent. Their performance varies when applied to different scenarios.

### Deep learning methods

To enhance image quality and efficiency, researchers have made significant strides by integrating convolutional neural networks (CNNs) and generative adversarial networks (GANs). These networks enable independent learning of image feature information, resulting in higher quality and more realistic image enhancement. LLNet [20] represent a pioneering application of deep learning in low-light image enhancement. It utilizes a deep neural network structure that employs stacked sparse denoising encoders and an end-to-end training mechanism. However, the enhanced images produced by this method often exhibit residual noise and excessive smoothing. MBLLEN [21] enhances images by extracting features at various levels through multiple sub-networks. While this approach improves the quality of enhanced images in several aspects, some outputs may exhibit a somewhat overexposed effect in certain cases. Wei et al. [22] introduced the Retinex Network (Retinex-Net), which integrates neural networks with Retinex theory to decompose images into reflectance and illumination components. The method learns the light-independent reflectance and the smoothness of the illumination map, followed by enhancement and denoising of both components. While this approach achieves clearer image enhancement, it is susceptible to random noise. Zhang et al. [23, 24] successively proposed the KinD and KinD++ algorithms for the illumination component that can be flexibly adjusted, using the same decomposition network as that of Retinex, and the enhancement and denoising processes can be carried out for the reflection and illumination components respectively on the basis of Retinex-Net. This method has better results in color recovery, but there is the problem of unclear local details.

In response to challenges in supervised learning, such as overfitting and the difficulty of obtaining paired images. Guo et al. [25] proposed a zero-learning method Zero-Reference Deep Curve Estimation (Zero-DCE). Zero-DCE tackles low-light image enhancement by framing it as a curve estimation problem. By treating a low-light image as input and generating higher-order curves as output, it adjusts the input dynamic range at the pixel level to produce an enhanced image. However, this method heavily relies on multiple exposure training data, neglects noise considerations, and is ineffective under extreme enhancement conditions. Jiang et al. [26] proposed Enlighten Generative Adversarial Network (EnlightenGAN), an unsupervised generative adversarial network. EnlightenGAN incorporates a global-local discriminator structure to capture more detailed features, coupled with self-regularized perceptual loss and attention mechanisms for enhanced results. Recently, Wu et al. [27] introduced URetinex-Net, a Retinex-based deep unfolding network. This approach reformulates the optimization problem into a learnable network, effectively addressing the decomposition problem by implicitly regularizing the model. Through adaptive fitting of the implicit prior in a data-driven manner, URetinex-Net achieves noise suppression and detail preservation in its decomposition results. Ma et al. [28] proposed a new self-calibrating illumination learning framework (SCI) is proposed that establishes a cascading illumination learning process with weight sharing to achieve image enhancement. A self-calibration module is constructed to reduce the computational cost and an additional network module is introduced to assist training to enable testing using only a single block, improving the efficiency of the model while enhancing the image quality. Hue et al. [29] proposed a novel unsupervised enhancement framework (PSENet) to address the limitations of current methods in dealing with overexposed images, which trains the network by constructing synthetic images to simulate all potential exposure scenarios, making it robust to various lighting conditions and allowing for better enhancement of images under extreme conditions. Fu et al. [30] proposed Learning a Simple Low-light Image Enhancer from Paired Low-light Instances (PairLIE), an unsupervised method for learning adaptive priors from pairs of shimmering images and designed a simple self-supervised mechanism to remove implausible features from the original images to assist Retinex decomposition. Two low-light images were utilized for training to fully extract the information from the low-light images, and a simpler network was utilized to achieve image enhancement. Wang et al. [31] proposed a new zero-reference low-light enhancement framework (QuadPrior), which is based on the physical light transfer theory and designs a light-invariant prior to connect normal-light images and low-light images. And a lightweight a priori image framework was designed to be trained using normal illumination images to automatically realize low light enhancement. Yu et al. [32] proposed a novel learning-based perceptual resampling method. This approach utilizes model knowledge to learn perceptual information from input images, enabling the customization of resampling features, which further enhances the model’s ability to extract features. Lv et al. [33] proposed a novel zero-shot framework called FourierDiff, which embeds Fourier priors into a pre-trained diffusion model to mitigate the degradation of the model’s capabilities. Moreover, this method has low requirements for training data. To produce better visual results, a spatial frequency optimization method was further designed to precisely enhance image detail, achieving superior enhancement outcomes. Zhu et al. [34] proposed a simple and efficient flow-based image enhancement framework, FlowIE, which estimates a direct path from feature distribution to high-quality images. A linear many-to-one transport mapping is constructed through conditional rectification to accelerate the network’s inference capability. Furthermore, a faster inference algorithm was introduced, optimizing path estimation using the tangent direction at the midpoint based on the Lagrange mean law, to achieve better visual results. Shi et al. [35] proposed a novel method that combines denoising and enhancement of low-light images, which is not affected by training data or noise. It adjusts the enhancement level of each pixel by scaling the denoised image based on the illumination intensity. Then, noise is removed from the original low-light image in the form of reflections, improving the network’s denoising capability. This approach achieves optimal enhancement results without losing image information.

In conclusion, both types of methods have their limitations in enhancing images. The former relies more on manual parameter adjustments, performing poorly in complex scenarios; while the latter relies heavily on extensive data support, requiring high-quality training data and necessitating precautions against overfitting. Although the existing models obtain good image results, there are still problems such as blurred details and poor denoising. The method in this paper deals with both detail preservation and denoising and gets better results.

### Proposed method

**Fig 2:**
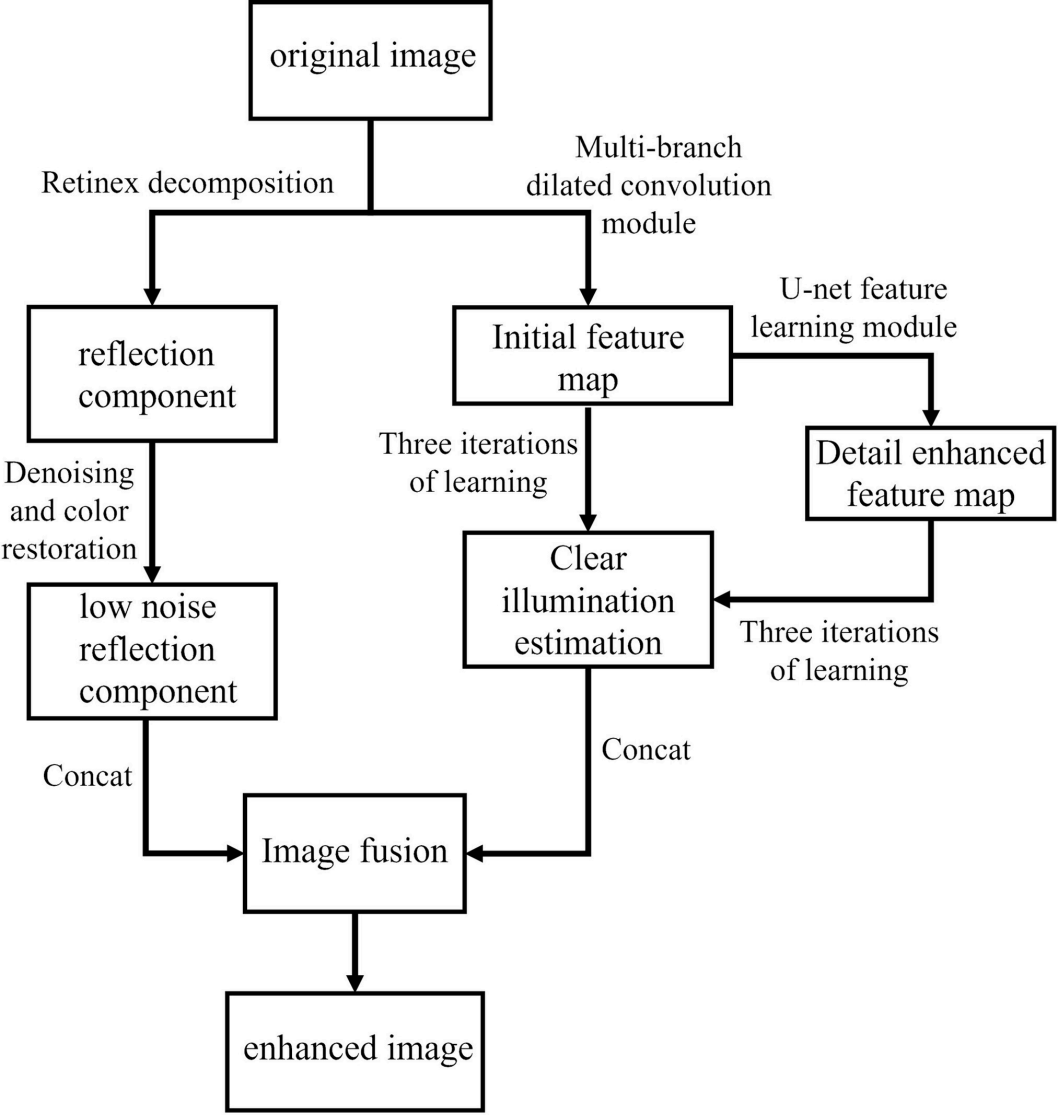
The framework of the proposed model.

The ILR-Net framework is divided into two branches: Retinex decomposition and feature enhancement, with the overall flowchart shown in Fig 2. The final enhanced image is obtained by merging the low-noise reflection component, which contains rich color signals from the Retinex decomposition, with the illumination estimation from the feature enhancement branch.

**Fig 3:**
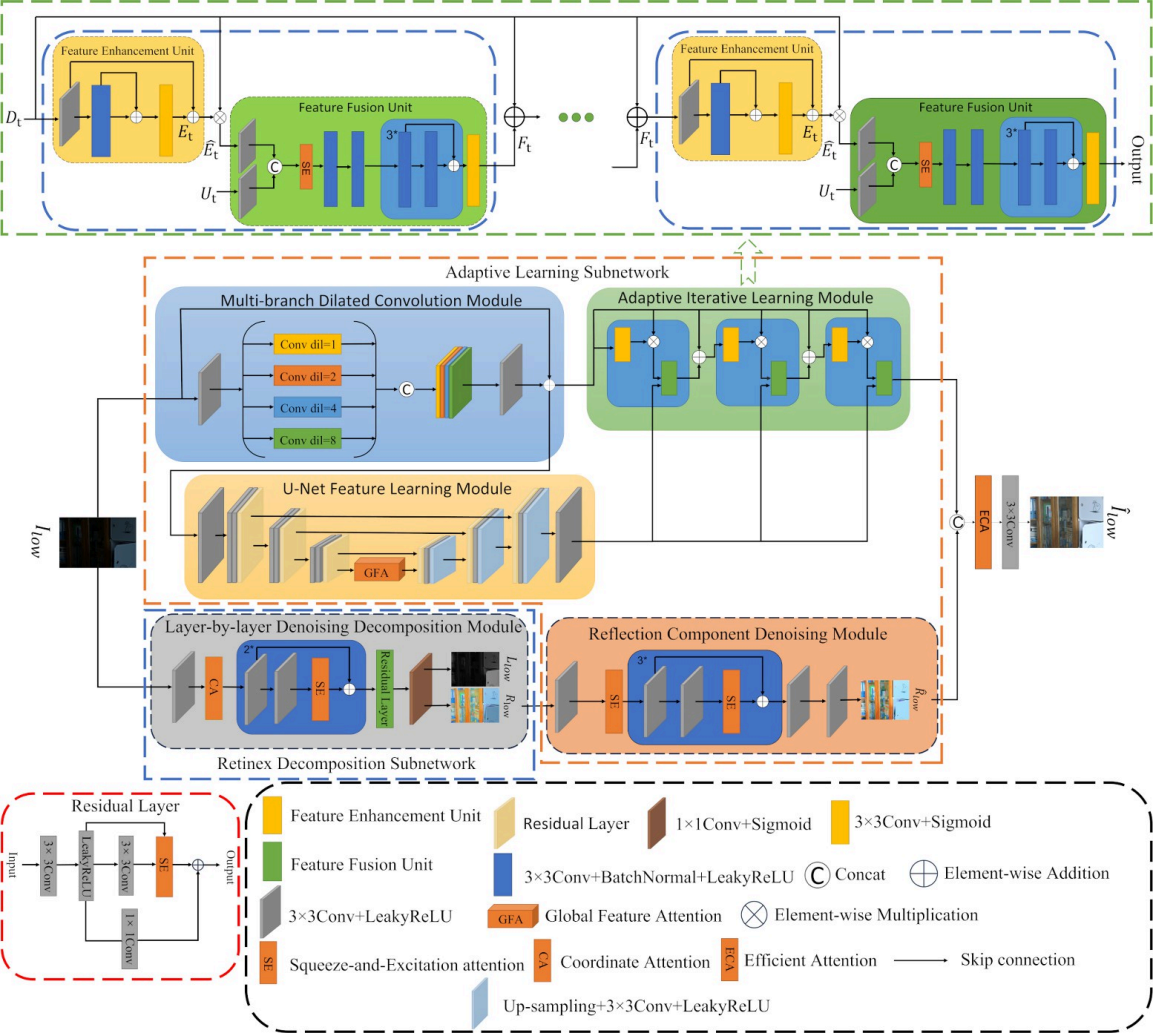
The framework of the proposed model.

ILR-Net network structure is shown in Fig 3. In the adaptive learning sub-network, the multi-branch dilation convolution module and the U-Net feature learning module perform feature extraction on the input image, respectively, and the results obtained from the former go through the feature enhancement unit for deeper feature learning. Subsequently, the resulting feature map is dot-multiplied with the results of the multi-branch dilation convolution module, and the resulting feature map and the results of the U-Net module go to the feature fusion unit for feature fusion to obtain a clearer image. In Retinex decomposition sub-network, the low light image is noise suppressed and decomposed into illumination component and reflection component based on the use of CA [14] and SE [15]. The obtained reflection component undergoes further feature extraction and denoising operations through the reflection component denoising module to obtain a low-noise clear reflection component. Finally, the clear image derived from the adaptive learning sub-network is spliced with the denoised reflection component on the channel, and the final enhanced image is obtained by Efficient Attention (ECA) [36] and 3×3 convolution.

### Multi-branch dilated convolution module

**Fig 4:**
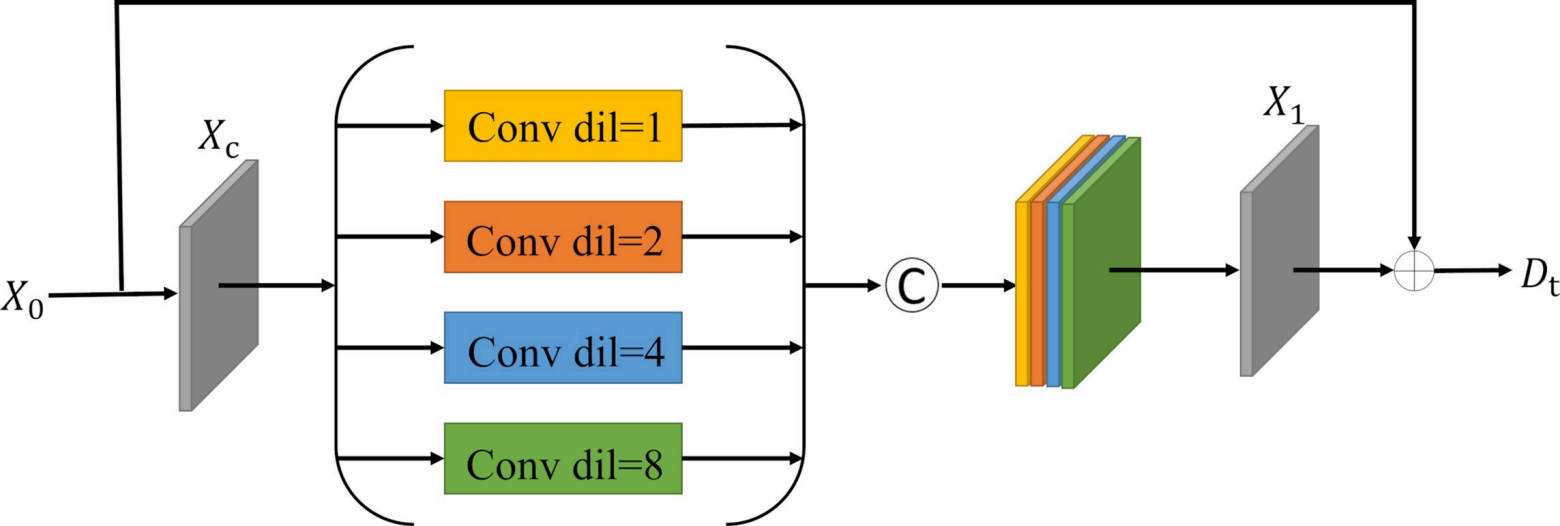
Multi-branch dilation convolution module.

The multi-branch dilation convolution module is shown in Fig 4. Taking the original low-light image as input, initial feature extraction is first performed by 3×3 convolution. Secondly, after four layers of dilation convolution [37] branches with expansion rates of 1,2,4,8 respectively is used for feature learning under different sensory fields. Finally, the feature maps of each branch are merged to obtain an image containing rich feature information. Dilation convolution can expand the receptive field of the network without using a large convolution kernel, thus obtaining richer features. However, due to the unique nature of dilation convolution, concatenating dilation convolutions with the same expansion rate will easily lead to discontinuous sampling features and grid effect [38]. Therefore, the expansion rate of the dilation convolution is set different ensure the continuity of the sampling and sensing fields. The whole computational process of the multi-branch dilation convolution module can be shown as follows:

$$\left\{\begin{array}{c} X_{c}=Conv_{3\times 3}\left(X_{0}\right)\\ X_{1}=Conv_{3\times 3}\left(\left[DC_{d=t}\left(X_{c}\right);\ldots \right]\right)\\ D_{t}=X_{0}+X_{1} \end{array}\right.$$ (2)

where *DC* stands for dilated convolution, *t*(t = 1,2,4,8) stands for dilation rate, and *X* stands for the corresponding feature map.

### U-net feature learning module

**Fig 5:**
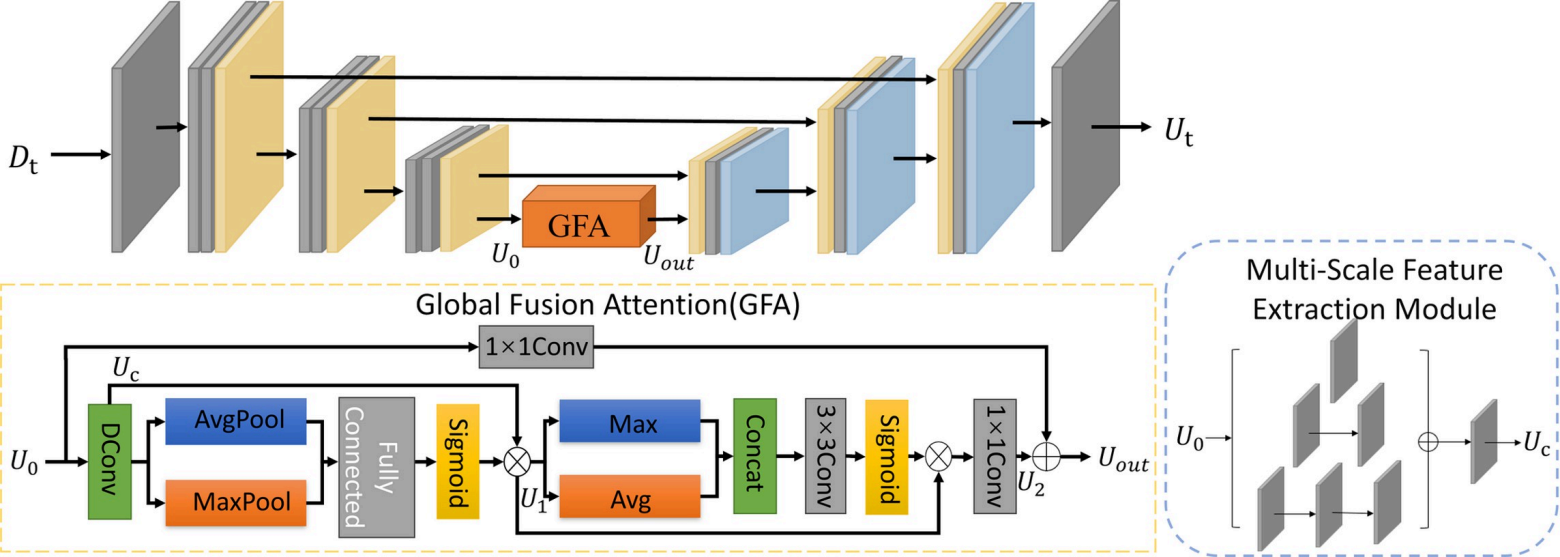
U-Net feature learning module.

To further extract rich detail information from low-light images, a U-Net module based on the full convolution strategy is designed on the basis of multi-branch dilation convolution and adds a residual layer in each layer in order to fuse more feature information. Inspired by CBAM [16], global feature attention (GFA) is designed. A multiscale feature extraction module is introduced in GFA, which extracts feature information from the input feature map using convolutional layers in parallel and fuses local and global feature information using residual structure. The multi-branch feature extraction module utilizes multiple 3×3 convolution kernels concatenated together instead of larger convolution kernels. These concatenated kernels are then incorporated into the network in a parallel manner to reduce parameter count and acquire rich feature information. The Fig 5 illustrates the structure of the U-Net feature learning module alongside the attention mechanism of the GFA.

Within the U-Net network, the lowermost layer’s small-resolution feature map holds extensive feature details. Hence, this paper integrates the GFA module into this layer to enhance feature learning within the network. Given an input feature U_0_, it first goes through the multibranch feature extraction module for feature extraction, then uses AvgPool2d and MaxPool2d to obtain the detail information, then goes through the fully connected layer and uses the Sigmoid function to normalize it in the interval 0–1, and then multiplies it by the result U_c_ of the multibranch feature extraction module for feature fusion to obtain the feature map U_1_. Subsequently, maximum pooling and average pooling are performed in the channel dimension, and after splicing, they are normalized using the Conv+Sigmoid function, and then multiplied by the feature map U_1_ to obtain the feature map U_2_. Finally, the original input U_0_ is added with the feature map U_2_ to realize the fusion process of the global feature information. The whole calculation process is shown below:

$$\left\{\begin{array}{c} U_{c}=Conv_{3\times 3}\left(U_{0}\right)+Conv_{3\times 3}\left(Conv_{3\times 3}\left(U_{0}\right)\right)+Conv_{3\times 3}\left(Conv_{3\times 3}\left(Conv_{3\times 3}\left(U_{0}\right)\right)\right)\\ U_{1}=\sigma \left(FC\left(AvgPool\left(U_{1}\right)\right)+FC\left(MaxPool\left(U_{1}\right)\right)\right)⊗U_{c}\\ U_{2}=Conv_{1\times 1}\left(\sigma \left(Conv_{7\times 7}\left(\left[MaxPool\left(U_{2}\right);AvgPool\left(U_{2}\right)\right]\right)\right)⊗U_{1}\right)\\ U_{out}=Conv_{1\times 1}\left(U_{0}\right)+U_{2} \end{array}\right.$$ (3)

where *FC* and *σ* denote the fully connected and Sigmoid activation functions.

### Adaptive iterative learning module

**Fig 6:**
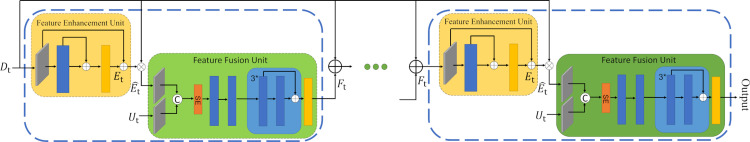
Adaptive iterative learning module.

The adaptive iterative learning module, shown in Fig 6, uses a fully convolutional network for adaptive learning and iterative convergence to obtain optimal results. It consists of two parts: a feature enhancement unit and a feature fusion unit. The feature map D_t_ that passes through the multi-branch dilation convolution module is input to the feature learning unit to get the enhanced feature map E_t_ and the input image D_t_ are subjected to Hadamard product operation to obtain the output result $\hat{E_{t}}$; The input of the feature fusion unit consists of $\hat{E_{t}}$ and the result U_t_ from the U-Net feature learning module. Firstly, the channels are spliced, and the SE enables the network to concentrate on learning the useful channel information, and then through the fusion unit based on Conv+BatchNormal+ReLU, it speeds up the fusion of the feature information and the convergence of the model, and obtains the converged feature mapping Ft. Subsequently, Ft is used as the input to the next stage of the loop, and the whole iterative learning process shares the weights, and finally a clear converged enhanced image is obtained. obtain a clear converged enhanced image.

#### Feature enhancement unit

The feature enhancement unit uses Conv+BatchNormal+ReLU for feature extraction and normalization of the input feature maps and improves the learning ability of the network through residual structures with a uniform convolution size of 3 × 3. BatchNormal normalizes each channel and reduces the dependency between channels to improve the generalization ability of the network. The first estimated enhancement component E_t_ is first obtained as the input to the feature fusion unit. The feature enhancement unit is computed as follows:

$$\left\{\begin{array}{c} f_{c}=Conv_{3\times 3}\left(D_{t}\right)\\ f_{1}=BN\left(Conv_{3\times 3}\left(f_{c}\right)\right)\\ f2=fc+f1\\ E_{t}=\sigma \left(Conv_{3\times 3}\left(f_{2}\right)\right)+D_{t} \end{array}\right.$$ (4)

where *BN* and *σ* denote the BatchNormal and Sigmoid activation functions, respectively.

#### Feature fusion unit

In order that the network does not lose the detailed features of the image during the learning process, so the input of the feature fusion unit consists of the result U_t_ of the U-Net feature extraction module and the result $\hat{E_{t}}$ of the multiplication of the output E_t_ and D_t_ of the feature enhancement unit, and the size of the convolution is also uniformly 3 × 3. Firstly, $\hat{E_{t}}$ and U_t_ are processed through the convolution layer for the feature extraction and spliced in the channel dimension, and the weights are assigned to their channels through the SE, and the processed results are K_c_ into the fusion unit. The fusion unit consists of two layers of Conv+BatchNorm+ReLU and three stacked network structures based on Conv+BatchNorm+ReLU and adopts the hopping connection to transfer the feature information. K_c_ is normalized by the fusion unit, and the corresponding feature mapping F_t_ is obtained through Conv + Sigmoid. The obtained result F_t_ is used as input for the next loop. The calculation process is shown below:

$$\left\{\begin{array}{c} K_{c}=\rho \left(\left[Conv_{3\times 3}\left(U_{t}\right);Conv_{3\times 3}\left(E_{t}\right)\right]\right)\\ K_{1}=BN\left(Conv_{3\times 3}\left(BN\left(Conv_{3\times 3}\left(K_{c}\right)\right)\right)\right.\\ K_{2}=K_{1}+BN\left(Conv_{3\times 3}\left(BN\left(Conv_{3\times 3}\left(K_{c}\right)\right)\right)\right.\\ K_{3}=K_{2}+BN\left(Conv_{3\times 3}\left(BN\left(Conv_{3\times 3}\left(K_{2}\right)\right)\right)\right.\\ K_{4}=K_{3}+BN\left(Conv_{3\times 3}\left(BN\left(Conv_{3\times 3}\left(K_{3}\right)\right)\right)\right.\\ F_{t}=\sigma \left(Conv_{3\times 3}\left(K_{4}\right)\right) \end{array}\right.$$ (5)

where *BN*、*σ* & *ρ* represent BatchNormal, Sigmoid activation function and SE, respectively.

### Layer-by-layer denoising decomposition module

**Fig 7:**
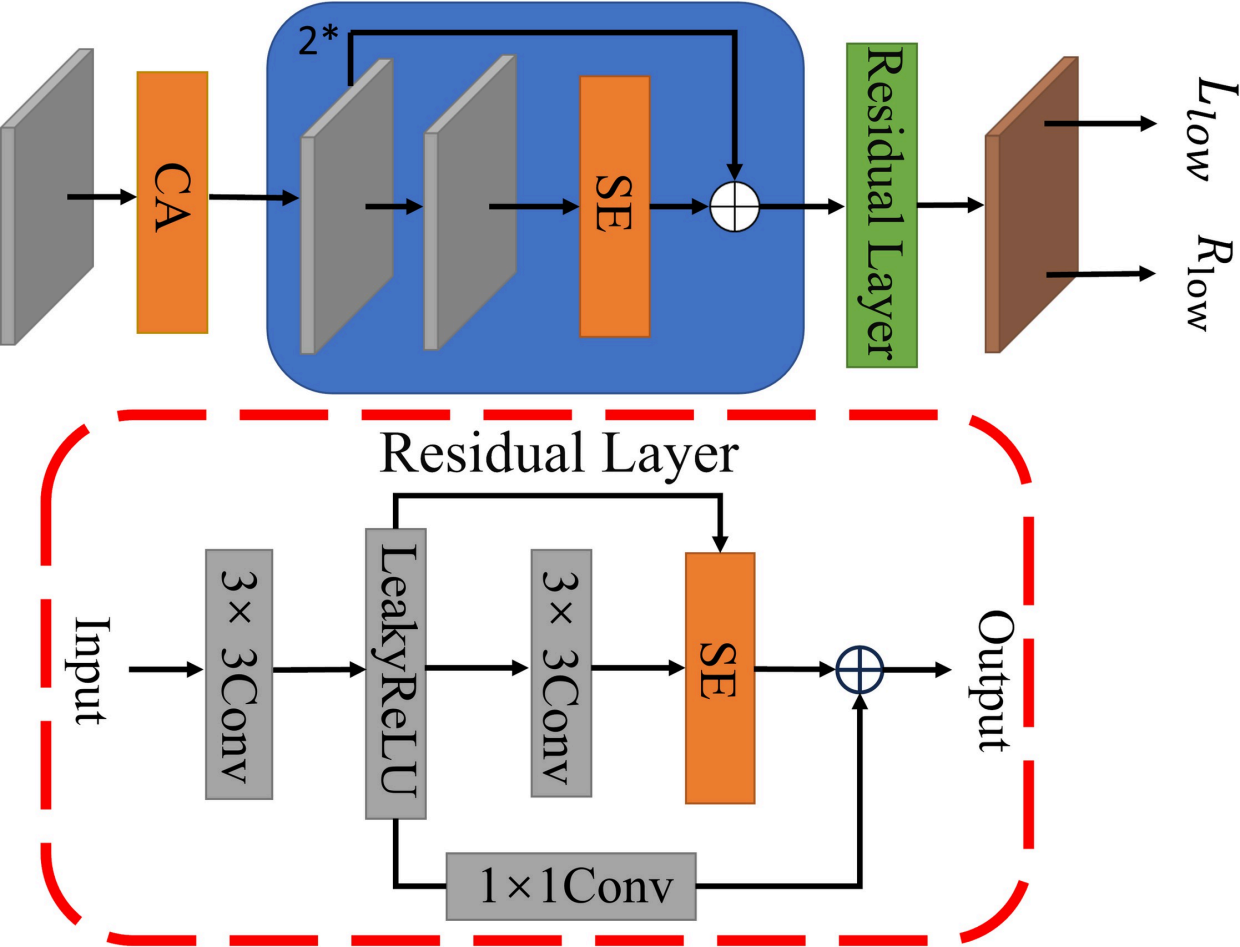
Layer-by-layer denoising decomposition module.

To minimize noise generated during the decomposition process and retain detailed image information, a layer-wise denoising decomposition module was devised based on CA and SE [14, 15]. This module shares parameters during the training process and the structure of the layer-by-layer denoising decomposition module are shown in Fig 7. Firstly, feature extraction is performed on the low-light image using 3×3 convolution. Second, CA is employed to allocate varying weights to the feature information within the feature map based on their coordinates. This assigns smaller weights to pixel coordinates with higher noise levels and larger weights to those with lower noise levels, thereby achieving noise suppression within the feature map. Subsequently, two on-channel denoising operations are performed to further suppress the noise by performing deeper feature extraction using two 3×3 Conv+ReLU for the shallow features from the previous stage and estimating the noise level in each channel by assigning weights to the channels of the feature map using the SE. Meanwhile, to enhance the detail retention capability of this module, the adjusted features are fused multiple times using a residual structure to prevent loss of detail information. Finally, the reflection and illumination components are decomposed using a residual block and a 3×3 convolution.

### Reflection component denoising module

**Fig 8:**
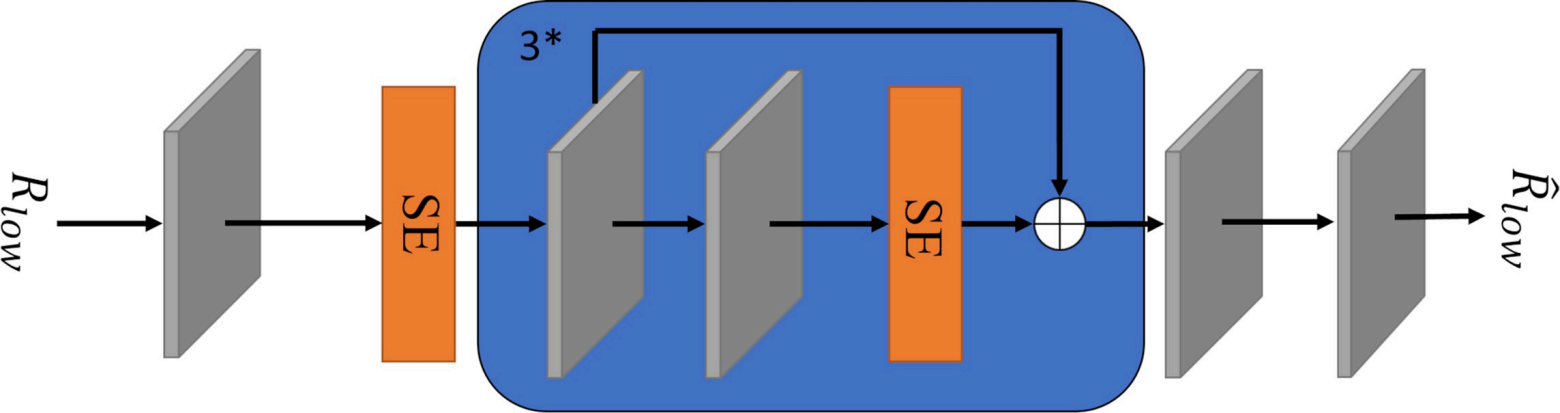
Reflection component denoising module.

Based on Retinex theory, the reflection component reflects the characteristics of the object and contains a lot of color detail information. To better deal with low light images, the noise and artifacts in the reflection component are reduced as much as possible. In this paper, a reflection component denoising module is designed to further suppress the noise in the reflection component after decomposition, to obtain a clear and detailed image. The structure of the reflection component denoising module is shown in Fig 8.

This module aims to fine-tune the initial reflection component *R*_*low*_ obtained from the layer-by-layer denoising decomposition module. Firstly, after a 3×3 convolution for initial feature extraction, the noise present in each channel is evaluated again by SE [15] and the corresponding weights are assigned. Secondly, deeper feature extraction of the feature maps obtained in the previous stage is performed after three iterations of a learning mechanism consisting of two Conv+ReLU layers and a SE, which uses a residual learning strategy so that the model focuses on learning the detailed information of the image. The final noise reduced image is then output after a Conv+ReLU layer and a 3×3 convolution. The convolution kernels are consistently sized at 3×3, and the fill mode is configured to copy, thereby preventing edge artifacts.

### Loss function

The loss function design in this paper is divided into two parts, adaptive learning sub-network and Retinex decomposition sub-network are trained separately.

#### Loss function of the adaptive learning sub-network

An unsupervised loss function has been devised to train the network, further taking into account the structural, spatial, and perceptual information of the image. This loss function can be expressed as:

$$L_{total}=\lambda_{CB}L_{CB}+\lambda_{Per\;}L_{Per}+\lambda_{SSIM\;}L_{SSIM}+\lambda_{Ref\;}L_{Ref}$$ (6)

where, *L*_*CB*_ denotes Charbonnier Loss (CB); *L*_*Per*_ denotes Perceptual Loss (Per); *L*_*SSIM*_ denotes Structural Similarity Loss (SSIM); *L*_*ref*_ denotes the reflection consistency loss; λ_*CB*_, λ_*per*_, λ_*SSIM*_ and λ_*ref*_ represent the corresponding loss coefficients in order to better balance each loss function and optimize the network performance. They are respectively set to 1.0,1.0,0.1,0.01.

Charbonnier Loss: Instead of the conventional L1 loss, the Charbonnier loss is adopted to approximate it, aiming to minimize the disparity between the enhanced image and the real image under genuine conditions. The Charbonnier loss proves to be more advantageous in optimizing the model and enhancing the performance of image processing tasks, especially when combatting noise, preserving edge information, or handling outliers. The formula for Charbonnier loss is presented below:

$$L_{CB}=\sqrt{\|y-\hat{y}{\|}^{2}+c}$$ (7)

where *y* and $\hat{y}$ denote the real image and the enhanced image under normal light conditions, respectively. The constant *c* regulates the rate of change of the loss function as it approaches zero, ensuring stability. In this paper, *c* is set to 10^−6^.

Perceptual Loss: The incorporation of perceptual loss in this paper addresses the issue of excessive smoothing caused by structural similarity loss. Perceptual loss measures image disparities by leveraging the intermediate representation of a pre-trained neural network model. This approach enhances the preservation of detailed information and visual fidelity, thereby augmenting the realism of the image. The formula for perceptual loss is provided below:

$$L_{Per}=\frac{1}{W_{i,j}H_{i,j}C_{i,j}}\cdot \sum_{x=1}^{W_{i,j}}\sum_{y=1}^{H_{i,j}}\sum_{z=1}^{C_{i,j}}\left\|\varphi_{i,j}{\left(\hat{y}\right)}_{x,y,z}-\varphi_{i,j}{\left(y\right)}_{x,y,z}\right\|$$ (8)

where *y* and *$\hat{y}$* denote the real image and the enhanced image under normal light conditions, respectively. *W*_*i*,*j*_ and *H*_*i*,*j*_ represent the height and width of the feature maps obtained from the ith block and jth convolution, respectively, while *C*_*i*,*j*_ denotes the channel. φ_*i*,*j*_ represents the feature maps acquired from the ith block and jth convolutional layer of the pre-trained Visual Geometry Group16 (VGG16) model.

Structural Similarity Loss: The Structural Similarity (SSIM) metric quantifies the likeness between images based on their brightness, contrast, and structural characteristics. It assesses the resemblance between the original image in standard lighting conditions and the improved version image to enhance the preservation of structural details and intricate features. The formula for computing the structural similarity loss is provided below:

$$L_{SSIM}(x,y)=1-\frac{\left(2\mu_{x}\mu_{y}+c_{1}\right)\left(2\sigma_{xy}+c_{2}\right)}{\left(\mu_{x}^{2}+\mu_{y}^{2}+c_{1}\right)\left(\sigma_{x}^{2}+\sigma_{y}^{2}+c_{2}\right)}$$ (9)

where *x* and *y* denote the test image and the reference image, respectively. *μ*_*x*_ and *μ*_*y*_ represent their respective mean values, reflecting the brightness information. $\sigma_{x}^{2}$ and $\sigma_{y}^{2}$ denote the variances of x and y, reflecting the contrast information. σ_*xy*_ signifies their covariance, reflecting the structural information of the image. Additionally, *c*_1_ and *c*_2_ are constants close to zero and non-zero, respectively, introduced to prevent division by zero issues.

Reflection consistency loss: different from the reflection similarity loss in the decomposition sub-network, this loss function measures the differences between images by comparing the differences between corresponding pixels or feature points. The reflection components are extracted separately for the input and output images, and then the squared Euclidean distance between these two reflection components is calculated as the loss value. The formula for the reflection consistency loss is shown:

$$L_{Ref}=\frac{1}{N}\sum_{i=1}^{N}{\left\|R_{low\;}^{\left(i\right)}-R_{high\;}^{\left(i\right)}\right\|}^{2}$$ (10)

where *N* represents the sum of pixel points; $R_{low\;}^{\left(i\right)}$ and $R_{high\;}^{\left(i\right)}$ represent the reflective component of the ith pixel point of the input low-light image and the enhanced image, respectively.

#### Loss function of the Retinex decomposition subnetwork

To retain the structural information of the original image and enhance the noise reduction capability of the decomposition sub-network, the loss function *L*_*Decom*_ of the decomposition sub-network can be expressed as:

$$L_{Decom}=L_{recon}+\alpha L_{ir}+\beta L_{smooth}$$ (11)

where *L*_*recon*_ denotes the decomposition reconstruction loss; *L*_*ir*_ denotes the reflection similarity loss; *Lsmooth* denotes the illumination smoothing loss; *α* and *β* denote the weighting coefficients of the different losses.

Decomposition reconstruction losses are expressed as:

$$L_{recon}=\sum_{low\;}\sum_{normal\;}{\left\|R_{low\;}\circ I_{normal}-S_{low\;}\right\|}_{1}$$ (12)

where *R*_*low*_ and *I*_*normal*_ denote the reflection component and illumination component obtained after decomposition, respectively; *S*_*low*_ denotes the original real image; $\cdot \|\circ {\|}_{1}$ is the L1 parameter.

The loss of reflective similarity is expressed as:

$$L_{ir}={\left\|R_{low}-R_{normal\;}\right\|}_{1}$$ (13)

where *R*_*low*_ denotes the reflected component of the low illumination image; *R*_*normal*_ denotes the reflected component of the original image under normal light conditions.

The illumination smoothing loss is denoted as:

$$L_{smooth}=\sum_{i=low,normal\;}\left\|\nabla I_{i}\circ \exp\left(-\lambda_{g}\nabla R_{i}\right)\right\|$$ (14)

where ∇*I*_*i*_ and ∇*R*_*i*_ denote the gradients of the illuminated and reflected components, respectively; and *λg* represents the weighting coefficients.

## Experimental results and analysis

### Experimental environment and training settings

We use PyTorch deep learning framework to conduct experiments, which are completed on Windows 10, Intel(R) i5-13600KF, NVIDIA GeForce 4070 GPU platform. During the training process, the training samples are uniformly adjusted to 600×400, and the training is performed on the public dataset LOL, and the Adam optimizer is used to optimize the model; the momentum parameters are set to β_1_ = 0.5, β_2_ = 0.999; the batch size (batch size) is set to 16; the number of iterative training times (epoch) is set to 300, and the first 200 times are set to the initial Learning rate (lr) = 0.001, in the next every 20 iterations after the end of training learning rate decay to 10% of the last.

### Image evaluation metrics

In this paper, we use widely used evaluation metrics to quantitatively evaluate the model effect, using peak signal-to-noise ratio PSNR, structural similarity (SSIM) [39], Multi Scale Structural Similarity (MS-SSIM) [40], Perceptual Image Quality Evaluator (PIQE) [41], Blind/Reference less Image Spatial Quality Evaluator (BRISQUE) [42], natural image quality evaluation (NIQE) [43] and learning to perceive image block similarity (LPIPS) [44]. There is no precise definition of PIQE and BRISQUE in terms of formulas, so this section will describe in detail the principles of calculating these two-assessment metrics without presenting mathematical formulas.

The peak signal-to-noise ratio can be expressed as:

$$\left\{\begin{array}{c} MSE=\frac{1}{H∙W}\sum_{i=0}^{H-1}\sum_{j=0}^{W-1}[X(i,j)-Y{\left(i,j)\right]}^{2}\\ PSNR=10log_{10}\left(\frac{{\left(2^{n}-1\right)}^{2}}{MSE}\right) \end{array}\right.$$ (15)

where H and W denote the length and width of the image, respectively; *X*(*i*,*j*) and *Y*(*i*,*j*) denote the test image and the reference image, respectively; and MSE denotes the mean square error. The larger value of PSNR represents the smaller distortion of the image, and the better quality of the image.

The structural similarity can be expressed as:

$$SSIM=\frac{\left(2\mu_{x}\mu_{y}+c_{1}\right)\left(2\sigma_{xy}+c_{2}\right)}{\left(\mu_{x}^{2}+\mu_{y}^{2}+c_{1}\right)\left(\sigma_{x}^{2}+\sigma_{y}^{2}+c_{2}\right)}$$ (16)

where x and y represent the test image and the reference image, respectively. *μ*_*x*_ and *μ*_*y*_ denote their mean values, while $\sigma_{x}^{2}$ and $\sigma_{y}^{2}$ represent their variances. *σ*_*xy*_ indicates their covariance, and *c*_1_ and *c*_2_ are non-zero constants introduced to prevent division by zero. Unlike PSNR, SSIM not only accounts for differences in brightness and contrast but also considers discrepancies in structural information, aligning more closely with human visual perception. SSIM ranges between 0 and 1, where values closer to 1 signify higher similarity between two images and better image quality. Both metrics offer insights into the degree of information preservation and the reconstruction quality of the enhanced image.

The natural image quality assessment can be expressed as:

$$NIQE=\sqrt{{\left(v_{1}-v_{2}\right)}^{T}{\left(\frac{\sum_{1}+\sum_{2}}{2}\right)}^{-1}(v_{1}-v_{2})}$$ (17)

where *v*_1_, *v*_2_, ∑_1_, and ∑_1_ represent the mean vector and covariance matrix of the natural MVG model and the distorted image Multivariate Gaussian (MVG) model, respectively. The MVG model is a multivariate Gaussian distribution model that can be used to describe the relationship between multiple variables that can be used in describing the distribution of pixels in a small area over the color and spatial domains and these feature vectors are used to compute the NIQE metric scores. The no-reference NIQE metric aligns more closely with human visual perception. A higher value indicates poorer image quality, while a lower value suggests greater similarity to the real image.

Learning to perceive image block similarity can be expressed as:

$$d\left(x,x_{0}\right)=\sum_{l}\frac{1}{H_{l}W_{l}}\sum_{h,w}{\left\|W_{l}⊙\left({\hat{y}}_{hw}^{l}-{\hat{y}}_{0hw}^{l}\right)\right\|}_{2}^{2}$$ (18)

where *d* represents the distance between *x* and *x*_0_. The feature stacks ${\hat{y}}_{hw}^{l}$ and ${\hat{y}}_{0hw}^{l}$ extracted at the L-layer are unit normalized in the channel dimension. The number of activated channels is then reduced using the vector *W*_*l*_, and the L2 distance is calculated. Finally, averaging over space and summing over channels is performed. Closer to subjective human perception, the lower the value of LPIPS, the smaller the perceived difference between two images.

Multi Scale Structural Similarity (MS-SSIM) can be expressed as:

$$MS-SSIM(x,y)={\left[l_{M}(x,y)\right]}^{\alpha M}\cdot \prod_{j=1}^{M}{\left[c_{j}(x,y)\right]}^{\beta j}{\left[s_{j}(x,y)\right]}^{\gamma j}$$ (19)

multi-scale approach to examine image details at different resolutions. The reference and distorted image signals are used as inputs, and a low-pass filter is applied iteratively to down-sample the filtered image by a factor of two. The resolution of the original image is assumed to be denoted as Scale1, and the resolution of the image after M-1 iterations is denoted as ScaleM. the contrast measure *c*_*j*_ (*x*,*y*) and the structure measure *s*_*j*_ (*x*,*y*) in SSIM are computed at the scales obtained in each iteration, and the luminance measure *l*_*M*_ (*x*,*y*) is computed only at the last scale, ScaleM. the composite metrics are obtained by synthesizing the results of the measurements at different scales. The indices *αj*, *βj*, and *γj* were used to adjust the different components, to simplify the choice of parameters and were set to *αj* = *βj* = *γj*.

Perceptual Image Quality Evaluator (PIQE): PIQE is an image quality evaluation algorithm based on human perception, which is capable of evaluating the quality of an image without the need of a reference image (without comparing the original image with the reference image). The principle of PIQE is that the image is divided into multiple chunks, and in each of the chunks some features related to human perception are computed, and these features are combined and subsequently. The block structure and noise features of the image are utilized to calculate the quality score of the image. The advantage of PIQE is that it can evaluate the quality of the image quickly and there is a good consideration of the influencing factors of human perception. The smaller the value of the result, the better the image quality is represented.

Blind/Reference-less Image Spatial Quality Evaluator (BRISQUE): The computational principle of the BRISQUE metric is to extract the mean subtracted contrast normalized (MSCN) coefficients from the image, fit the MSCN coefficients to an asymmetric generalized Gaussian distribution (AGGD), extract the features of the fitted Gaussian distribution, and input them into the support vector machine SVM to do the regression, so as to get the result of the image quality assessment. The smaller the value of the result, the better the image quality is represented.

### Comparison experiments

To assess the efficacy of the method proposed in this paper, we compare its experimental results with those of classical advanced image enhancement methods now. These include Retinex-Net (2018-BMVC) [22], KinD (2019-ACMMM) [23], EnlightenGAN [26], RRDNet (2020-ICME) [45], Zero-DCE (2020-CVPR) [25], Zero-DCE++ [46], RUAS (2021-CVPR) [47], SCI (2022-CVPR) [28], URetinex-Net (2022-CVPR) [27], UNIENet (2022-ECCV) [48], PSENet (2023-WACV) [29], PairLIE (2023-CVPR) [30], QuadPrior (2024-CVPR) [31] and subjective on seven datasets. visual comparison. On the quantitative side, the image quality of different methods is evaluated by seven image assessment metrics.

**Fig 9:**
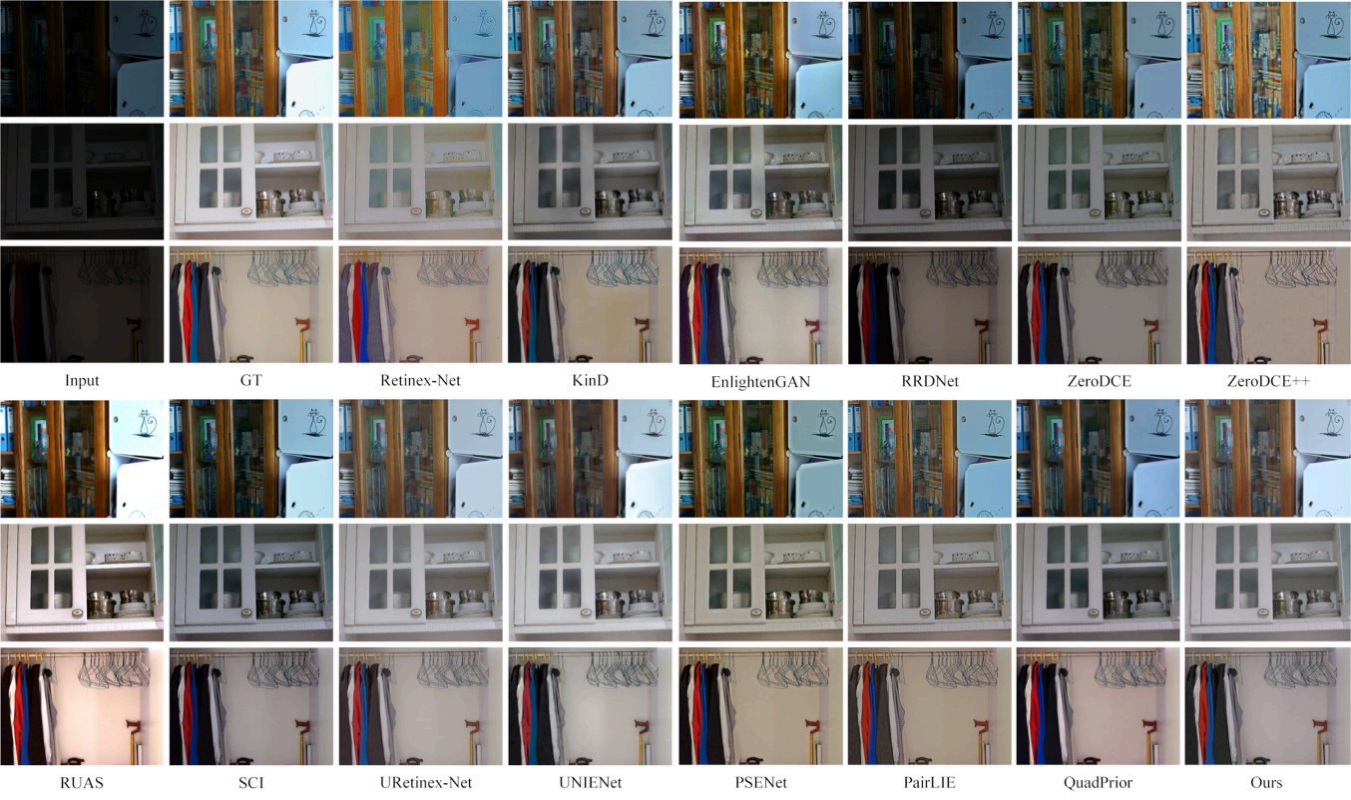
Subjective visualization of various methods on the LOL dataset.

As shown in Fig 9, the enhancement results on the LOL dataset [22] indicate that the Retinex-Net method captures the overall feature information of the image but introduces significant noise and color bias issues. The KinD method effectively removes noise but at the cost of losing some detail, resulting in an overly smoothed image. The RUAS method, while enhancing the image, suffers from overexposure, leading to an unnatural subjective appearance. Both the RRD-Net and SCI methods preserve the image’s color information well, but their enhancement of fine details is insufficient. PSENet, UNIENet, and PairLIE retain most of the image’s details while enhancing it, producing smoother visuals; however, their enhanced images tend to appear darker. Meanwhile, the EnglightenGAN, Zero-DCE, and Zero-DCE++ methods introduce excessive noise during enhancement. QuadPrior and URetinex-Net perform well in terms of image enhancement, noise reduction, and detail preservation. However, compared to these methods, the approach proposed in this paper achieves more natural results, particularly in restoring the original image’s color, resulting in a more visually pleasing and realistic outcome.

On the quantitative side, as shown in Table 1. It is lower than URetinex-Net method on SSIM metrics and ranks second. It lags behind EnglightenGAN, UNIENet in NIQE index. It is worth noting that this paper’s method achieves better scores in PSNR, MS-SSIM, and LPIPS, which reach 23.7624, 0.8804, and 0.1583, respectively. It shows that this paper’s method has an overall advantage in terms of noise suppression, detail retention, and enhancement effect.

**Table 1:** Objective evaluation results of different algorithms on LOL datasets.

Methods		PSNR↑	SSIM↑	MS-SSIM↑	NIQE↓	LPIPS↓
**2018-BMVC**	**Retinex-Net [22]**	15.0246	0.7424	0.7313	5.3014	0.2830
**2019-ACMMM**	**KinD [23]**	17.9907	0.8153	0.8628	4.8625	0.2711
	**EnglightenGAN [26]**	17.8754	0.8040	0.8370	4.7410	0.3903
**2020-ICME**	**RRDNet [45]**	11.5934	0.4923	0.7936	4.8368	0.2532
**2020-CVPR**	**ZeroDCE [25]**	13.8509	0.7092	0.8362	7.8286	0.2605
	**ZeroDCE++ [46]**	15.0475	0.7638	0.8251	4.8593	0.1921
**2021-CVPR**	**RUAS [47]**	18.1165	0.6405	0.8237	4.9702	0.2989
**2022-CVPR**	**SCI [28]**	17.5441	0.5906	0.8603	8.4739	0.2397
**2022-CVPR**	**URetinex-Net [27]**	22.9581	**0.8723**	0.8747	5.0868	0.1761
**2022-ECCV**	**UNIENet [48]**	22.7347	0.8141	0.8372	**4.3235**	0.2419
**2023-WACV**	**PSENet [29]**	15.7821	0.7669	0.8523	8.1114	0.2621
**2023-CVPR**	**PairLIE [30]**	17.0092	0.8506	0.8360	4.9307	0.2239
**2024-CVPR**	**QuadPrior [31]**	20.1017	0.8085	0.8619	4.9023	0.1891
**Ours**		**23.7624**	0.8653	**0.8804**	4.7939	**0.1583**

**Fig 10:**
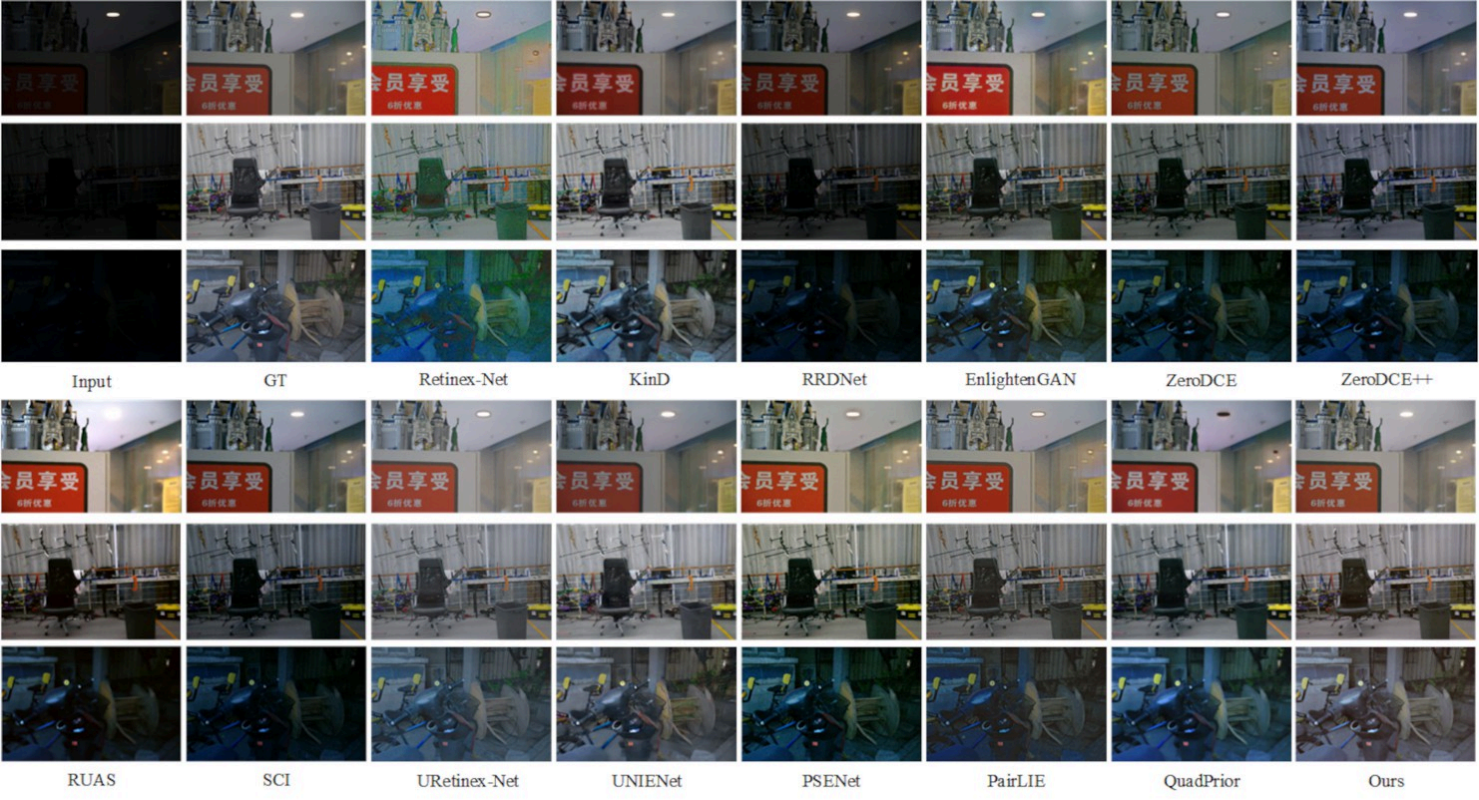
Subjective visualization of various methods on the LOLv2-Real dataset.

Further, this paper conducts experiments on the LOLv2-Real dataset [49], which contains 100 pairs of real low-light images, to better evaluate the performance of this paper’s method in real scenes. As shown in Fig 10, when handling indoor low-light environments, Retinex-Net introduces significant noise and color distortion. EnlightenGAN, RRDNet, ZeroDCE, ZeroDCE++, RUAS, and SCI also show poor enhancement results, with varying degrees of noise. The enhanced images produced by PSENet and PairLIE exhibit noticeable color bias compared to the real images. In contrast, KinD, UNIENet, URetinex-Net, QuadPrior, and the method proposed in this paper achieve more natural enhancement effects and improved visual quality. Similarly, in real night environments, most methods, except for KinD, UNIENet, URetinex-Net, and our approach, fail to produce noticeable enhancement. However, the KinD method tends to lose significant detail, resulting in overly smoothed images. In contrast, the UNIENet, URetinex-Net and this paper methods produce better results visually and the processed images look more realistic.

Also on the quantitative side, as shown in Table 2. Our method slightly lags behind URetinex-Net in the NIQE and LPIPS metrics, ranking second. However, this paper’s method outperforms all other index parameters, reaching 26.8252, 0.7784 and 0.8604 on PSNR, SSIM and MS-SSIM metrics, respectively. better demonstrating the applicability of this paper’s method on the LOLv2-Real dataset [49], which achieves better visual results in both indoor low-light environments and nighttime environments.

**Table 2:** Objective evaluation results of different algorithms on LOL-v2-Real datasets.

Methods		PSNR↑	SSIM↑	MS-SSIM↑	NIQE↓	LPIPS↓
**2018-BMVC**	**Retinex-Net [22]**	18.2339	0.4052	0.6850	4.5119	0.5268
**2019-ACMMM**	**KinD [23]**	23.9671	0.6549	0.8110	3.4996	0.3310
	**EnglightenGAN [26]**	17.8002	0.5792	0.8088	3.7574	0.4222
**2020-ICME**	**RRDNet [45]**	11.3911	0.3836	0.5672	3.8936	0.4686
**2020-CVPR**	**ZeroDCE [25]**	13.4027	0.3480	0.7467	3.5931	0.4210
	**ZeroDCE++ [46]**	13.3429	0.3734	0.4867	3.4836	0.4807
**2021-CVPR**	**RUAS [47]**	12.9074	0.3012	0.6533	3.6936	0.5410
**2022-CVPR**	**SCI [28]**	12.1603	0.3529	0.6492	3.6337	0.4298
**2022-CVPR**	**URetinex-Net [27]**	26.1457	0.7462	0.8502	**2.9793**	**0.2110**
**2022-ECCV**	**UNIENet [48]**	25.4989	0.7633	0.7837	3.9223	0.3762
**2023-WACV**	**PSENet [29]**	14.5476	0.3404	0.7877	8.8742	0.4169
**2023-CVPR**	**PairLIE [30]**	17.4583	0.6111	0.7202	4.3719	0.3570
**2024-CVPR**	**QuadPrior [31]**	19.7096	0.5461	0.8195	4.5765	0.3253
**Ours**		**26.8252**	**0.7784**	**0.8604**	3.1532	0.2522

**Fig 11:**
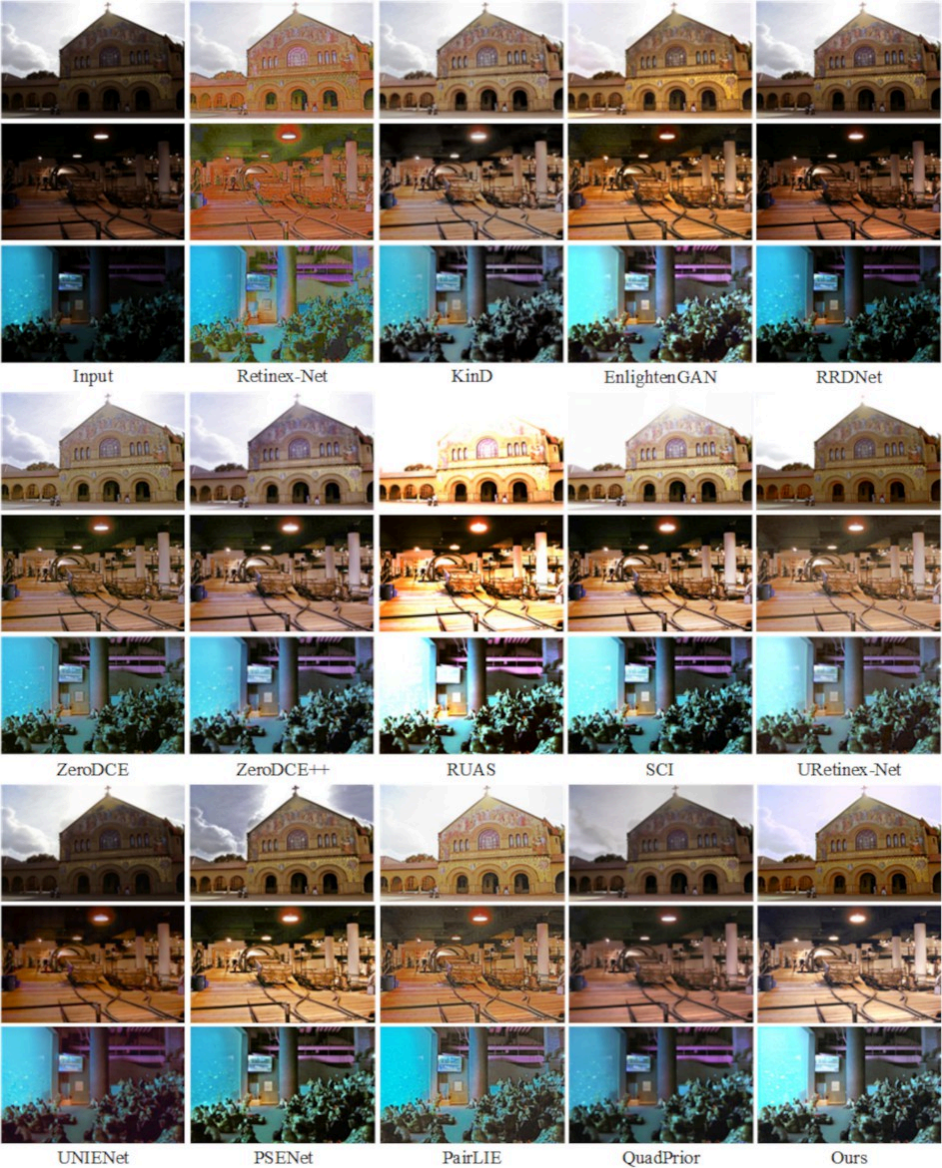
Subjective visualization of various methods on the DICM dataset.

**Fig 12:**
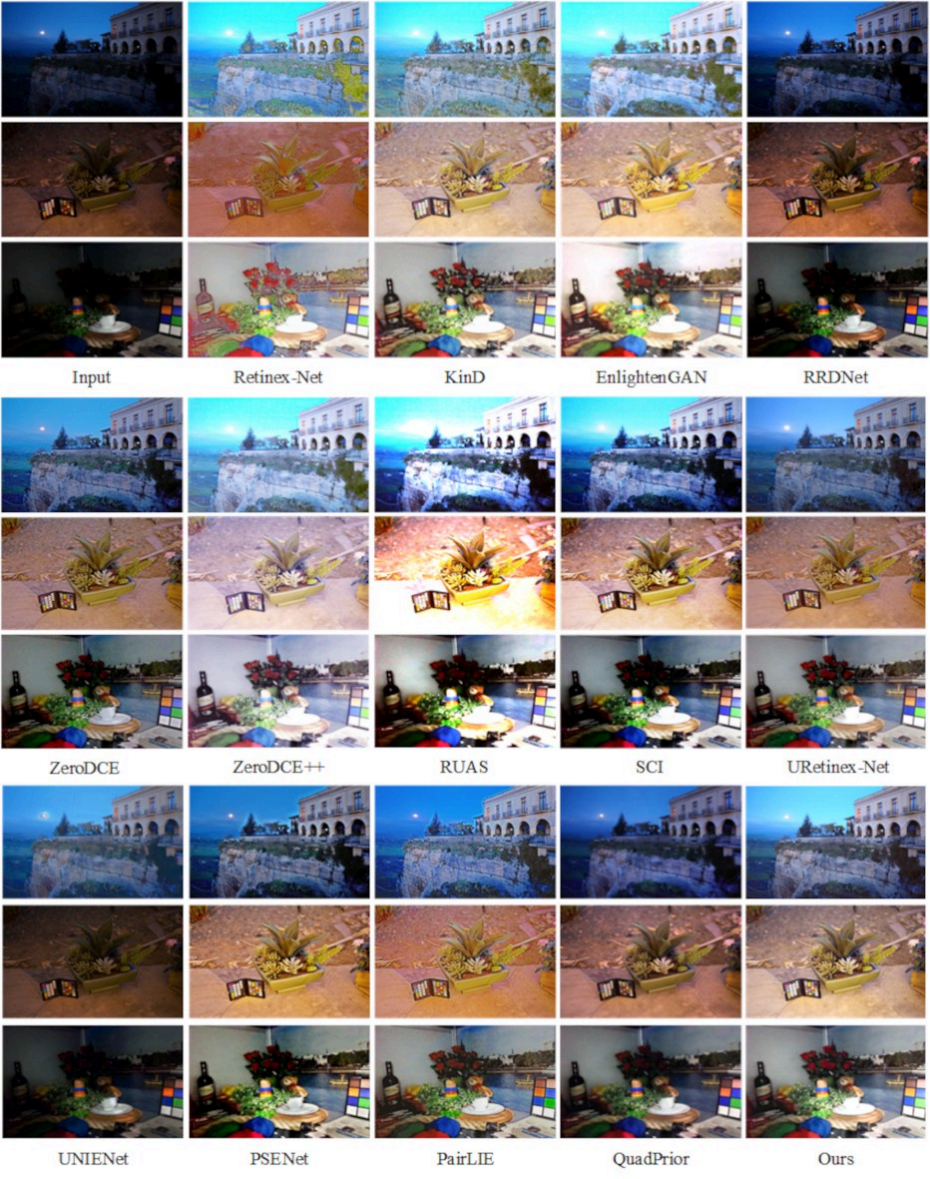
Subjective visualization of various methods on the MEF dataset.

**Fig 13:**
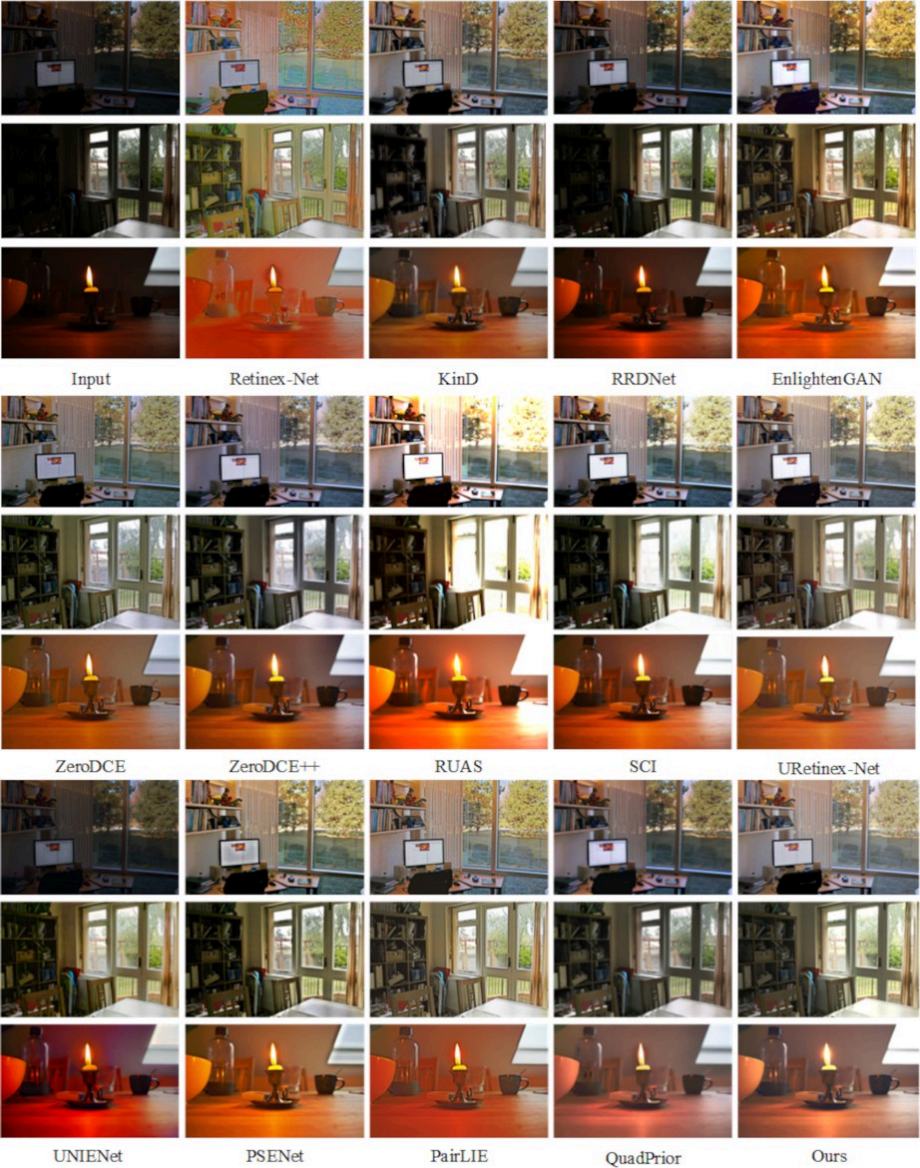
Subjective visualization of various methods on the LIME dataset.

**Fig 14:**
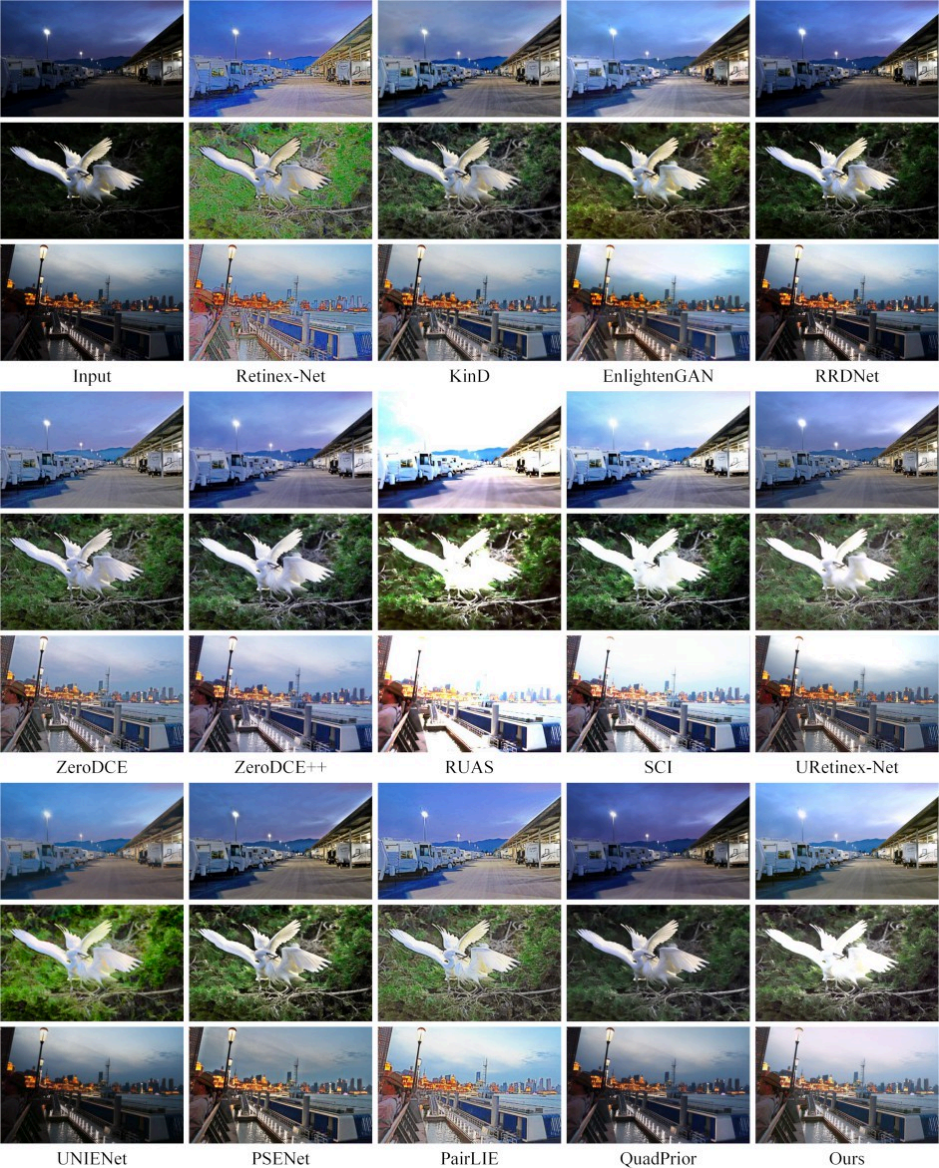
Subjective visualization of various methods on the NPE dataset.

**Fig 15:**
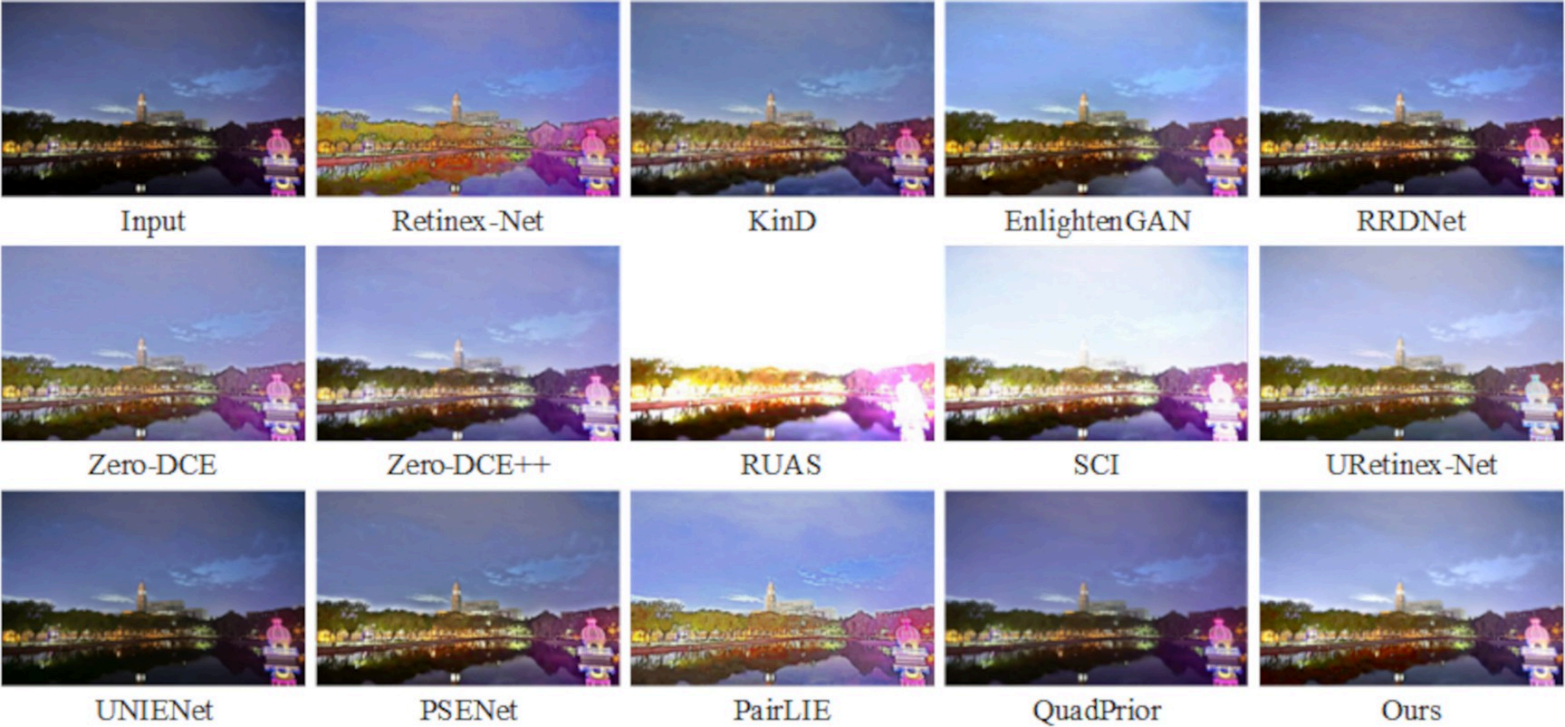
Subjective visualization of various methods on the Real-world dataset.

To assess the generalization capability of this paper’s method, experiments were conducted on five reference-free datasets DICM [50], MEF [51], LIME [52] and NPE [53], and the realistic shooting dataset Real-world, and the experimental results are presented in Figs 11–15.

Analyzing the enhancement results across these five datasets. On the DICM dataset, the Retinex-Net method showed a lot of noise and color distortion. The RRDNet, ZeroDCE, ZeroDCE++ and UNIENet methods showed little enhancement effect and lost a lot of detail. The PSENet and QuadPrior methods showed a lot of loss of detail and color shifting. EnlightenGAN, RUAS, SCI, URetinex-Net and PairLIE methods were overexposed, with much detail information lost, and individual methods showed varying degrees of noise. On the MEF, LIME, NPE, and Real-world datasets, the RRDNet, ZeroDCE, UNIENet, PSENet, PairLIE, and QuadPrior methods are able to retain the detailed information of the image better, but the overall color of the enhanced image is lighter, and the enhancement effect is not obvious enough. The Retinex-Net method still has a large amount of noise, serious artifacts and colour shifts, and the overall visual effect of the image is not natural enough. EnlightenGAN, ZeroDCE++, RUAS, SCI, URetinex-Net and PairLIE methods retain the original color information of the image, but all of them have different degrees of exposure and lose some details while enhancing. Although the method proposed in this paper also encounters exposure issues on the DICM and MEF datasets, it offers a more natural overall enhancement of the visual effect. In comparison, it effectively preserves the original detail information of the images, making the results more visually appealing.

On the quantitative side, as shown in Table 3. The method in this paper achieves higher scores on DICM, LIME, MEF, NPE and Real-world datasets, which further proves that our method also achieves better results on unpaired datasets compared to other frontier methods. The results of visual comparison and quantitative evaluation confirm that the images enhanced by the proposed method are closest to the real images, and close results are also obtained for unpaired low light images.

**Table 3:** Objective evaluation results of different algorithms on DICM, LIME, MEF, NPE, Real-world datasets.

Methods	DICM	LIME	MEF	NPE	Real-world
	NIQE↓/BRISQUE↓/PIQE↓	NIQE↓/BRISQUE↓/PIQE↓	NIQE↓/BRISQUE↓/PIQE↓	NIQE↓/BRISQUE↓/PIQE↓	NIQE↓/BRISQUE↓/PIQE↓
**Retinex-Net [22]**	3.1036/31.8683/29.9872	2.6292/13.9568/32.5578	4.1466/43.2680/49.3597	3.0583/26.1784/47.9425	3.8278/34.2726/44.1406
**KinD [23]**	2.5163/31.1606/42.1578	2.6108/14.2832/28.2970	3.6133/47.0684/55.9027	3.4134/25.5620/46.7214	3.7920/36.4782/41.1785
**EnglightenGAN [26]**	2.6392/27.6866/34.0548	2.7962/14.2252/26.4480	3.5265/35.9385/57.3759	2.9361/26.7796/42.7762	3.3622/33.4179/41.3934
**RRDNet [45]**	1.7577/29.8820/31.6983	2.8231/23.6603/32.8281	3.3962/52.8104/45.8889	2.9520/27.5569/43.1421	3.5789/32.6636/42.1240
**ZeroDCE [25]**	2.2824/23.9831/27.7152	2.7289/13.7444/28.0559	3.7191/49.4492/47.5915	2.8581/25.1417/41.3676	3.4557/35.5369/42.8659
**ZeroDCE++ [46]**	2.4626/25.2181/31.8866	2.6299/14.2218/25.3700	3.4060/33.7853/59.9575	2.8189/25.8055/40.7522	3.8561/35.5823/41.0167
**RUAS [47]**	4.4284/36.1740/55.6101	2.8572/33.7595/30.8190	3.6851/45.2590/65.4988	3.7678/36.7573/62.6978	4.8555/49.3987/49.5381
**SCI [28]**	3.1275/43.2270/34.0193	2.7816/14.9877/29.1212	3.1148/49.6955/55.8279	2.9413/27.1303/43.4285	3.7776/37.3542/47.4276
**URetinex-Net [27]**	4.6720/44.6266/30.9261	2.3106/13.7338/30.3740	2.9292/43.3036/44.4263	**2.6315**/27.2667**/**41.0804	3.4190/26.2348/37.4710
**UNIENet [48]**	4.1522/46.5513/37.9547	2.9125/19.6653/31.5548	3.7952/43.2496/66.4913	3.5048/35.6990/65.6545	3.3735/40.3308/53.8301
**PSENet [29]**	2.0163/29.8668/34.0084	2.5827/21.7540/25.3692	3.3662/32.4678/53.9009	2.7519/30.6916/**37.6078**	3.6208/30.4591/42.1407
**PairLIE [30]**	3.0626/45.3349/38.6633	2.5633/21.6813/28.8257	5.7968/42.4865/68.6092	3.0142/28.5879/45.1055	3.4119/26.8214/41.5817
**QuadPrior [31]**	3.4952/**16.3196/**29.6673	3.5525/25.3471/**19.0602**	3.6380/30.7356/48.9273	3.1896/31.6014/39.8193	4.1167/31.7236/**33.7291**
**Ours**	**1.7251/**20.3270/**21.6113**	**2.2942**/**13.0032**/21.6381	**2.8226**/**29.7337**/**43.8164**	2.6850/**24.0318**/38.3943	**3.1751**/**22.7590**/37.7874

### Ablation experiments

In order to verify the effectiveness of each module and loss function in this paper’s method, this subsection carries out ablation experiments on the LOL dataset [22] for the model and the joint loss function, respectively, and carries out the network and loss function changes according to the configurations in Tables 2 and 3 (√ stands for the module and loss function that have not been removed), and each incremental and decremental network, loss function weights, and parameter settings of the training are kept unchanged. PSNR and SSIM are used to comprehensively evaluate the image quality in terms of brightness, structural contrast, and noise.

#### Network module ablation experiments and analysis

For the network module ablation experiments, this subsection removes or partially deletes the Multi-branch Dilation Convolution Module (MDC), U-Net Feature Learning Module (U-Net), Reflection Denoising Module (Ref), Global Feature Attention (GFA), and Layer-by-layer Denoising Decomposition Module (Demo). The experiments have the following six combinations: ① H1: Only remove the multi-branch dilation convolution module and keep the others unchanged. ②H2: Only remove the U-Net feature learning module, replace it with the output of the feature enhancement unit, input it to the feature fusion unit for training, and keep the others unchanged. H3: Remove only the reflection denoising module and keep the rest unchanged. ④H4: Remove only the global feature attention and keep the others unchanged. ⑤H5: Use four-layer convolution instead of layer-by-layer denoising decomposition module for Retinex decomposition, other keep unchanged. ⑥H6: Remove both the initialization module and the U-Net feature learning module, others remain unchanged.

**Fig 16:**
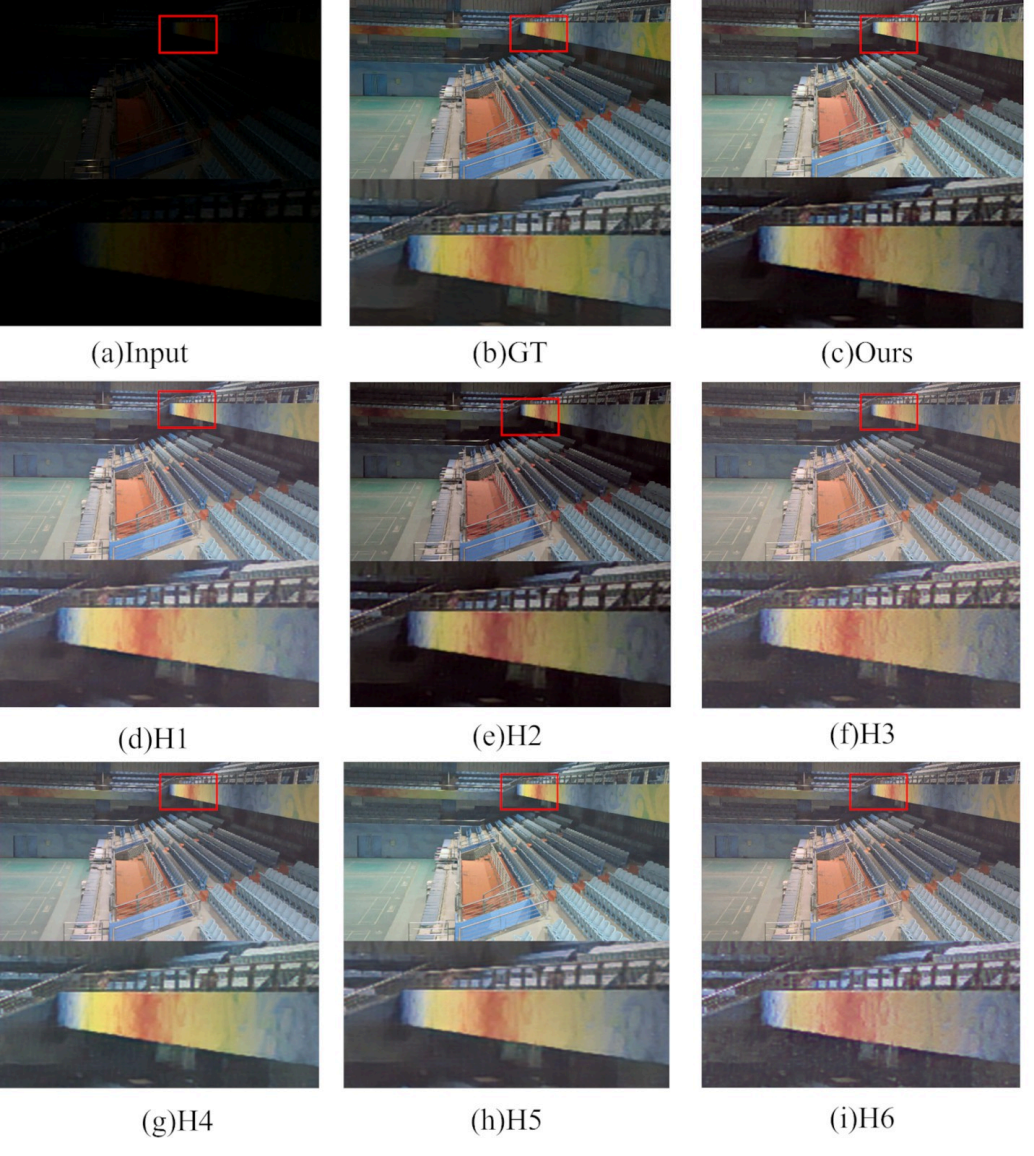
Subjective visualization of network module ablation experiments.

The subjective visual map of the network module ablation experiment is shown in Fig 16 with the details zoomed in to demonstrate the details. From the figure, the overall color information of the image after enhancement using H1 combination is light, and the color details are blurred. The image after enhancement using the combination of H2 loses part of the color information and noise appears around it. The enhanced image using the combination of H3 also has different degrees of noise and color deviation problems. The images enhanced with the combination of H4 and H5 lose more image texture details and show different degrees of noise and artifacts. And the image after enhancement using the combination of H6 lost serious detail information and showed serious color deviation, noise, and distortion problems.

In terms of quantitative aspects, the changes in the evaluation indexes after the removal of each module are shown in Table 4, from which the images enhanced with the combination of H1, H2, and H4 have a slight decrease in PSNR and SSIM indexes. The image enhanced with the combination of H3 and H5 has a significant decrease in the values of the two indicators, which reflects that both the reflection denoising module and the layer-by-layer denoising decomposition module have a significant effect on the denoising of the decomposed reflection map and restore the rich color information of the original image to the maximum extent. The image index after using H6 combination enhancement decreased seriously, in the absence of the original image initialization denoising and feature extraction, the adaptive iterative learning module is ineffective in noise suppression and detail retention, reflecting the necessity of the two modules, MDC and U-Net.

**Table 4:** Objective evaluation results of network module ablation experiments.

Module	MDC	U-Net	Ref	GFA	Demo	PSNR↑	SSIM↑
**H1**		√	√	√	√	21.1573	0.8266
**H2**	√		√	√	√	21.1861	0.8258
**H3**	√	√		√	√	20.7261	0.8081
**H4**	√	√	√		√	21.3070	0.8297
**H5**	√	√	√	√		21.7962	0.8301
**H6**			√	√	√	20.3357	0.8054
**Ours**	√	√	√	√	√	**21.8393**	**0.8315**

#### Adaptive learning subnetwork loss function ablation experiments and analysis

To perform ablation experiments for adaptive learning subnetwork loss function, this subsection removes or replaces the Charbonnier loss (*L*_*CB*_), the structural similarity loss (*L*_*SSIM*_), the perception loss (*L*_*Pre*_), and the reflection consistency loss (*L*_*Ref*_). The experiment has the following four combinations: ① L1: only *L*_*CB*_ is removed. ② L2: only *L*_*SSIM*_ is removed. ③ L3: only *L*_*Pre*_ is removed. ④ L4: only *L*_*Ref*_ is removed.

**Fig 17:**
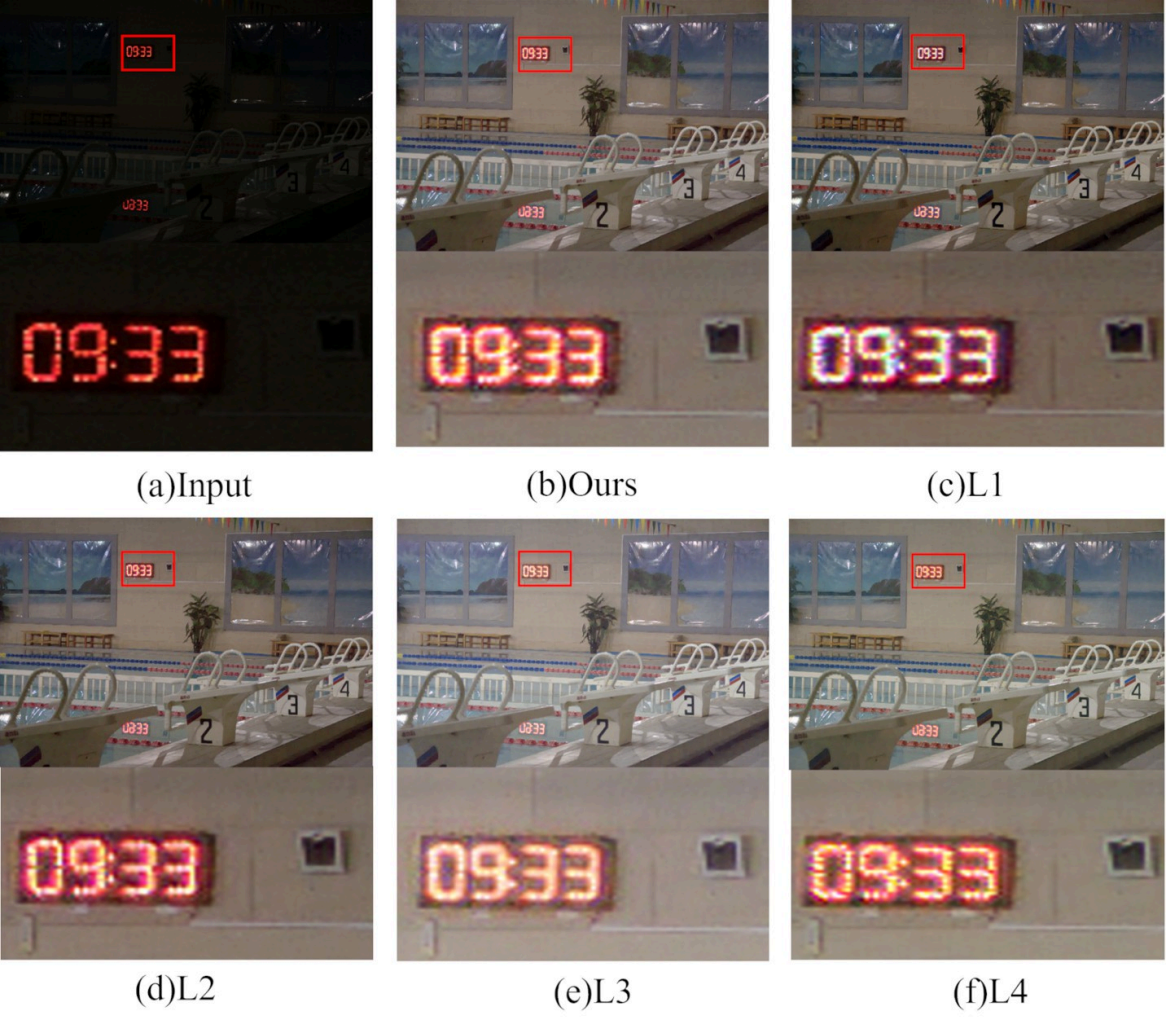
Subjective visualization of the loss ablation experiment.

The subjective visual representation of the loss function ablation experiment is depicted in Fig 17, with detailed sections enlarged for clarity. From the figure, it is apparent that the color information in images enhanced using the L1 combination is altered, and the image edges appear blurred. Images enhanced with the L2 and L4 combinations exhibit varying degrees of distortion and considerable noise. The combination using L3, on the other hand, lost more image texture details and showed different degrees of noise with off-color whitening.

Regarding quantitative aspects, changes in evaluation indices after removing each loss function are presented in Table 5. It is evident from the table that whether a certain loss function is removed or replaced, the objective evaluation indices PSNR and SSIM decrease compared to those in the method proposed in this paper, indicating the effectiveness of each loss function.

**Table 5:** Results of objective evaluation of loss ablation experiments.

Module	*L*_*CB*_	*L*_*SSIM*_	*L*_*Ref*_	*L*_*Pre*_	PSNR↑	SSIM↑
**L1**		√	√	√	22.3245	0.8620
**L2**	√		√	√	21.4777	0.8569
**L3**	√	√		√	20.1624	0.8425
**L4**	√	√	√		20.3633	0.8258
**Ours**	√	√	√	√	**22.7624**	**0.8653**

### Hyperparametric experiments with multibranch dilation convolution modules

**Fig 18:**
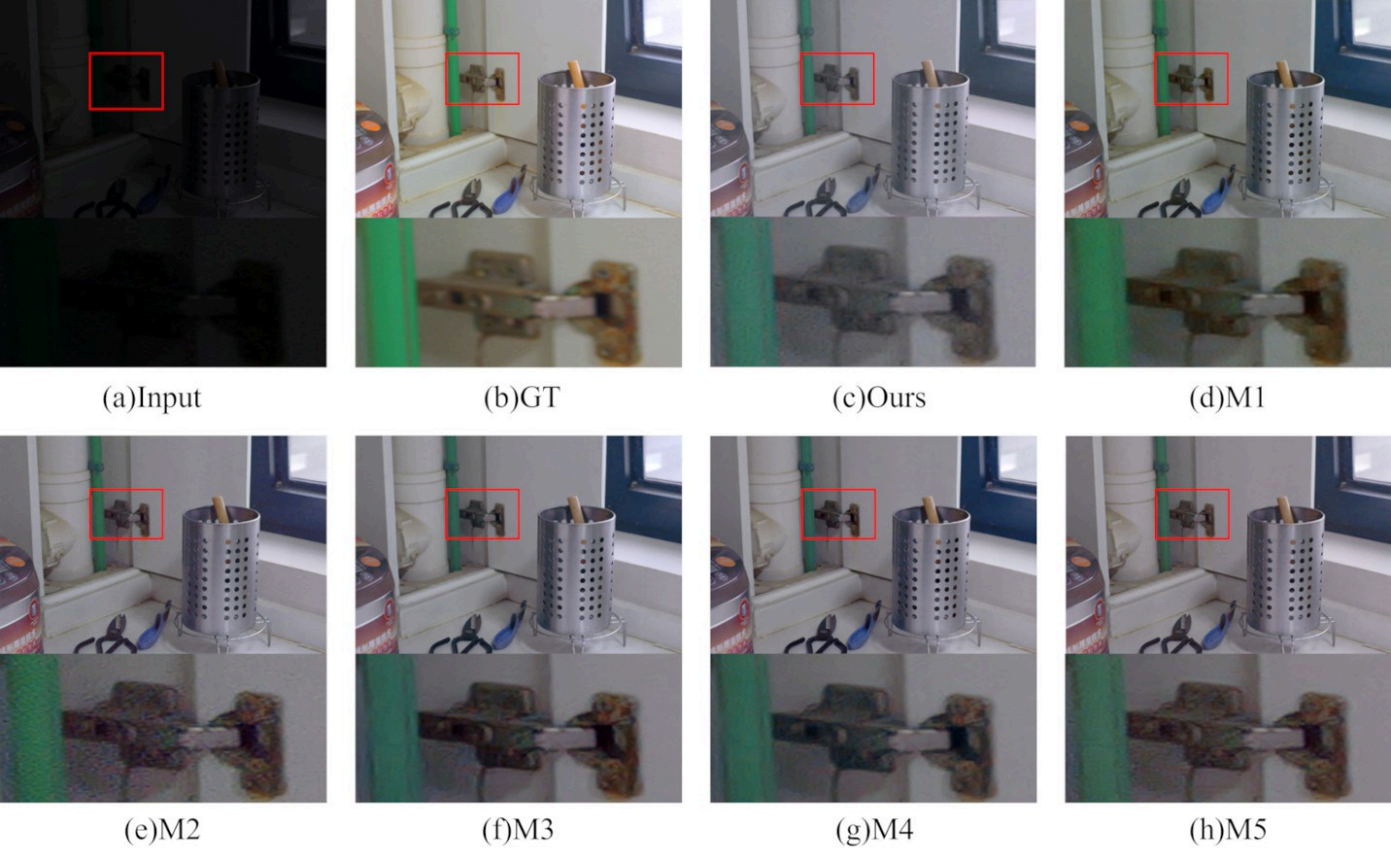
Multibranch dilation convolution modules ablation experiment subjective visual map.

To verify the usefulness of the choice of replacing the standard convolution with a dilation convolution and setting the number of convolution layers to 4 in the multibranch dilation convolution modules of this paper, test experiments were conducted on the LOL dataset [22] for this module. Firstly, the expansion rate of all the dilation convolutions in this module are set to 1 (M1) for the experiment. Secondly, experiments are conducted on models with the number of layers 1 (M2), 2 (M3), 3 (M4) and 5 (M5) in turn. Finally, comparison is made with the models in this paper. The subjective visualization after image enhancement is shown in Fig 18 with zoomed in details.

The figure illustrates that the brightness enhancement from the M1 model is subtle, with noticeable artifacts in the detailed areas. On the other hand, the image enhanced with the M2 model exhibits significant noise, accompanied by edge blurring. Enhancing with the M3 model results in varying degrees of color distortion. Images enhanced with the M4 and M5 models closely resemble the results obtained with the model proposed in this paper in terms of subjective perception. To further validate the model’s effectiveness, objective evaluations are conducted using two metrics, PSNR and SSIM, with comparison results presented in Table 6.

**Table 6:** Objective evaluation results of ablation experiments for multibranch dilation convolution module.

Module	PSNR↑	SSIM↑
**M1**	18.9496	0.8534
**M2**	17.8819	0.8169
**M3**	18.6650	0.8460
**M4**	19.4844	0.8576
**M5**	19.9776	0.8619
**Ours**	**22.3537**	**0.8670**

From the table, the use of dilation convolution can effectively improve the PSNR and SSIM values of the image. As the number of concatenated layers increases, the PSNR and SSIM values gradually increase, and the best score is obtained when the present algorithm (M4) is reached. When increasing to five layers (M5), the metric values decrease, and the model performance starts to degrade. Therefore, this module selects four layers of concurrent dilation convolution for feature extraction to achieve the best results.

### Limitations

**Fig 19:**
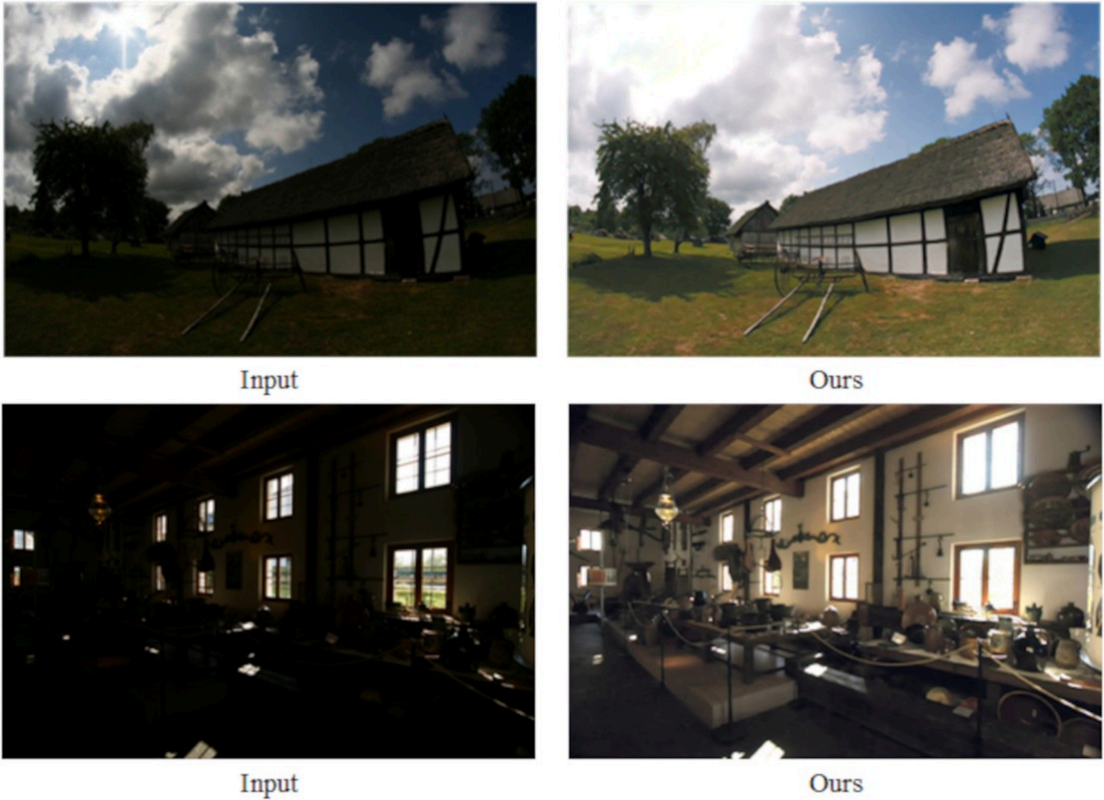
The visual effect of our method in enhancing images with both very dark and exposed areas.

In performing our tests, we found that our method lost a lot of detail information when processing images with both extremely dark and exposed areas, as shown in Fig 19. As can be seen from the window details in the figure and the area of the sun in the sky, our method is effective in enhancing brightness and retaining more color and detail information when processing low or medium brightness areas in an image, achieving better visual results. However, when dealing with overexposed regions, our method tends to over-enhance the brightness of this region, resulting in a serious loss of image details.

The reason for this problem is that our network does not limit the dynamic range of the brightness of the exposed area very well. When there are both very dark and exposed regions in the image, the network favors the enhancement of the darker regions. In addition, we failed to limit the enhancement strength of the exposed regions, and over-enhanced them to the point where the brightness of the exposed regions exceeded the brightness range of the image, losing a significant amount of detail.

In summary, our method can effectively enhance the overall brightness of an image and retain a large amount of detail in low-light and nighttime environments. However, we recognize the need to improve our method to achieve better enhancement when dealing with images with extremely dark and exposed regions. In our next work, we will focus on exploring ways to better limit the extent of exposure area enhancement to address this limitation, and to improve image quality by retaining more image details when dealing with images with both dark and exposed areas.

## Conclusions

This paper presents ILR-Net. The network comprises an adaptive learning sub-network and a Retinex decomposition sub-network. In the adaptive sub-network, initial feature extraction is conducted on the input low light image by concatenating dilation convolutions with varying expansion rates. The output results undergo deeper learning via the feature enhancement unit and the U-Net feature learning module. Subsequently, the feature fusion unit combines these results to generate the corrected enhanced image. The Retinex decomposition sub-network employs the Retinex theory to decompose the original image into light and reflection components. Noise generated during decomposition is suppressed multiple times to prevent detail loss from subsequent noise reduction. The reflection component is then denoised and enhanced using the reflection denoising module. Finally, the feature maps from both branches are concatenated in the channel dimension to produce the final enhanced image. The experimental results show that the method in this paper effectively improves the brightness of the image and recovers the details and color information of the image. It shows good visual results on seven datasets; it also gets higher scores on objective evaluation metrics. On the LOL and LOLv2-Real datasets, compared to the URetinex-Net and QuadPrior methods, our approach improved PSNR by 3.5%, 18.21%, and 11.10%, 36.10%, respectively, and improved MS-SSIM by 0.65%, 2.15%, and 1.20%, 4.99%, respectively. This further demonstrates the superiority of the proposed method. In our subsequent work, we will investigate combining the method with other computer vision domains and reducing the network size to be applied in more scenarios.

## Supporting information

S1 File (ZIP)
