# Supplementary figures and images for: ILR-Net: Low-light image enhancement network based on the combination of iterative learning mechanism and Retinex theory
Source: PLoS One. 2025 Feb 13;20(2):e0314541. doi: 10.1371/journal.pone.0314541 (PMC11825054; doi:10.1371/journal.pone.0314541)

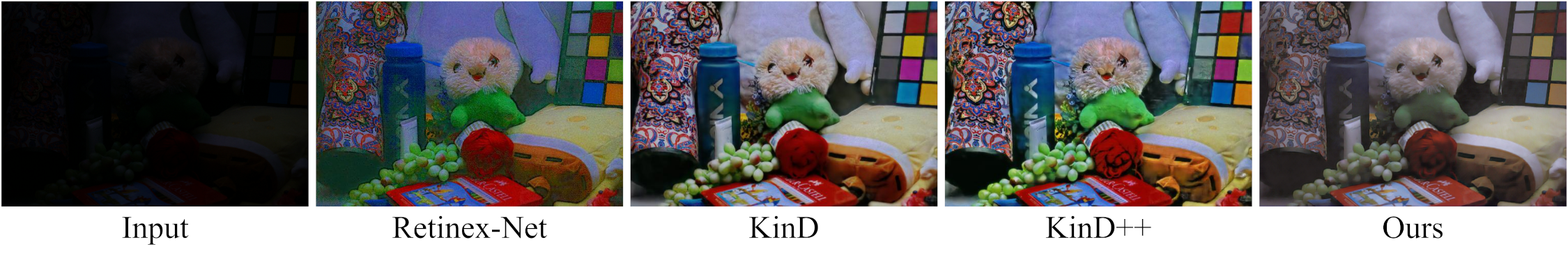

Supplement: S1 File — (ZIP) [file pone.0314541.s001.zip › Supporting Information/Fig 1.tif]

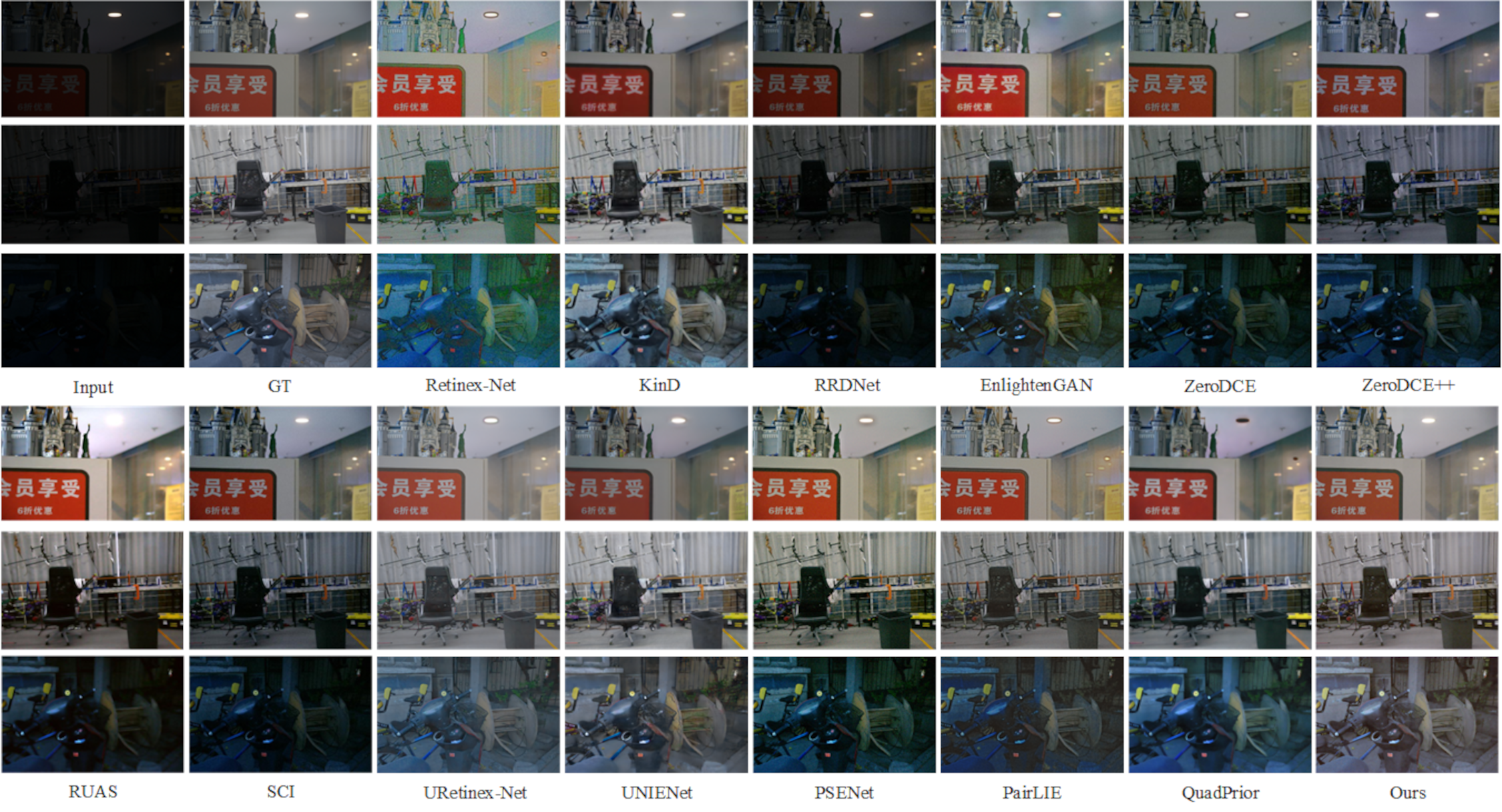

Supplement: S1 File — (ZIP) [file pone.0314541.s001.zip › Supporting Information/Fig 10.tif]

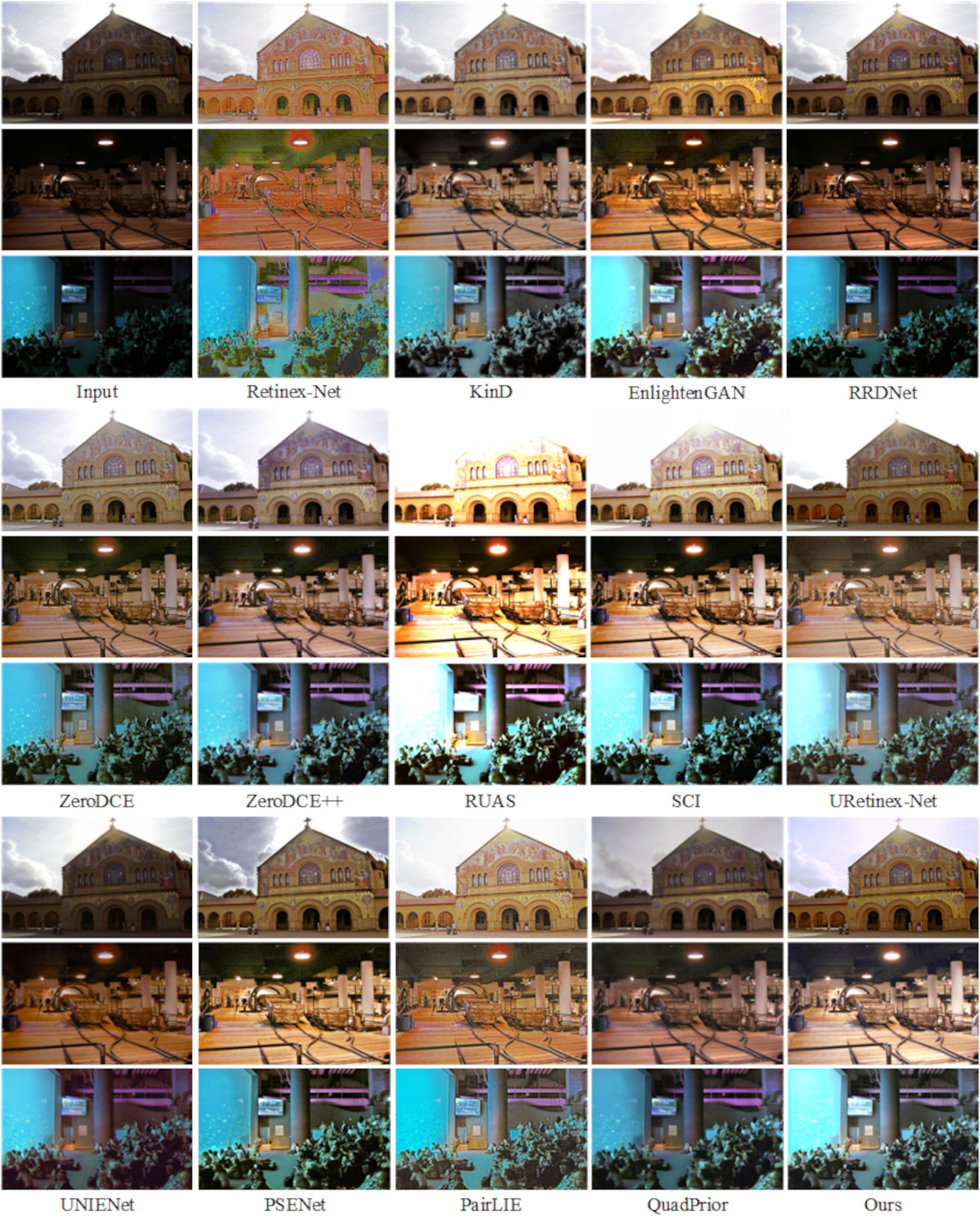

Supplement: S1 File — (ZIP) [file pone.0314541.s001.zip › Supporting Information/Fig 11.tif]

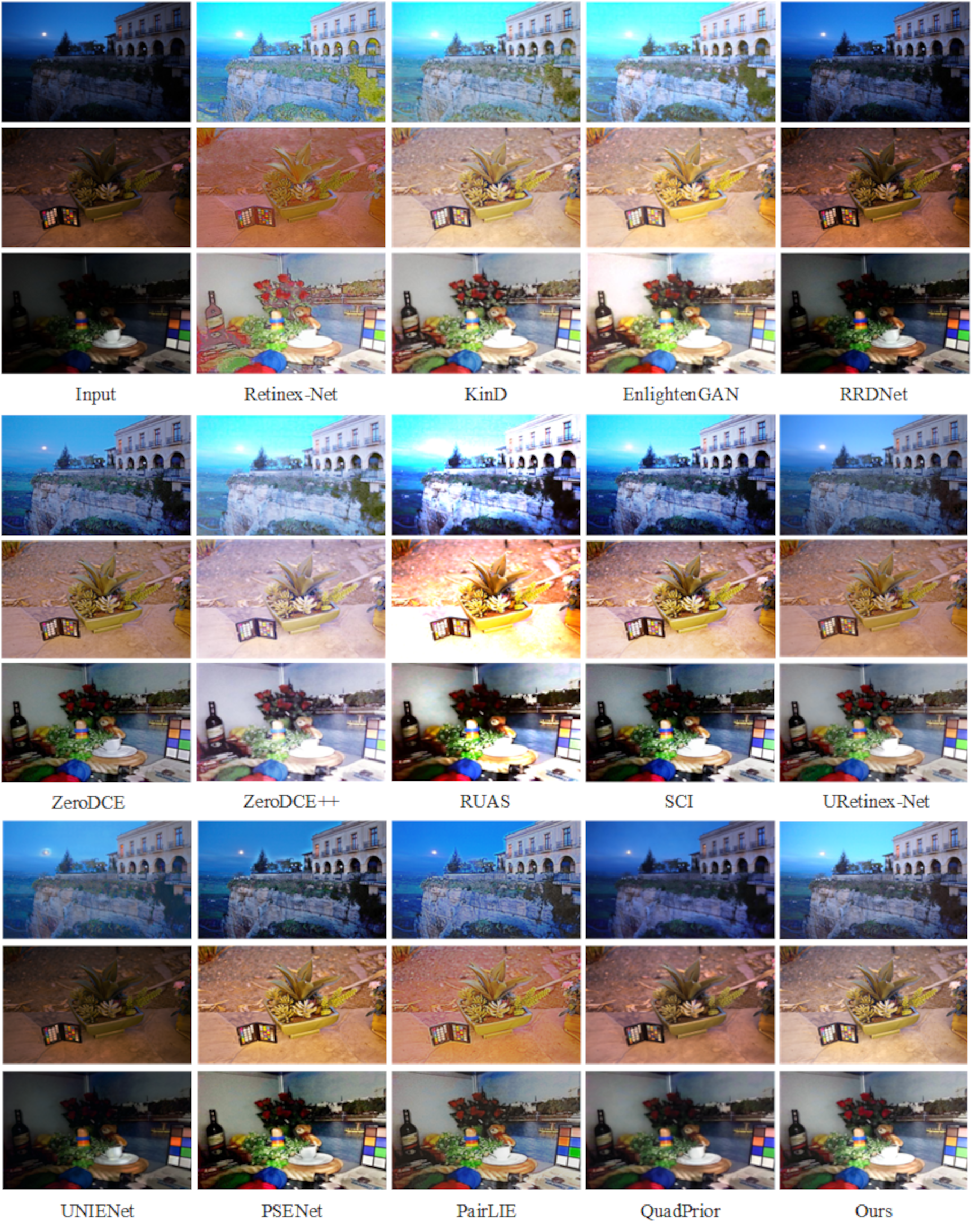

Supplement: S1 File — (ZIP) [file pone.0314541.s001.zip › Supporting Information/Fig 12.tif]

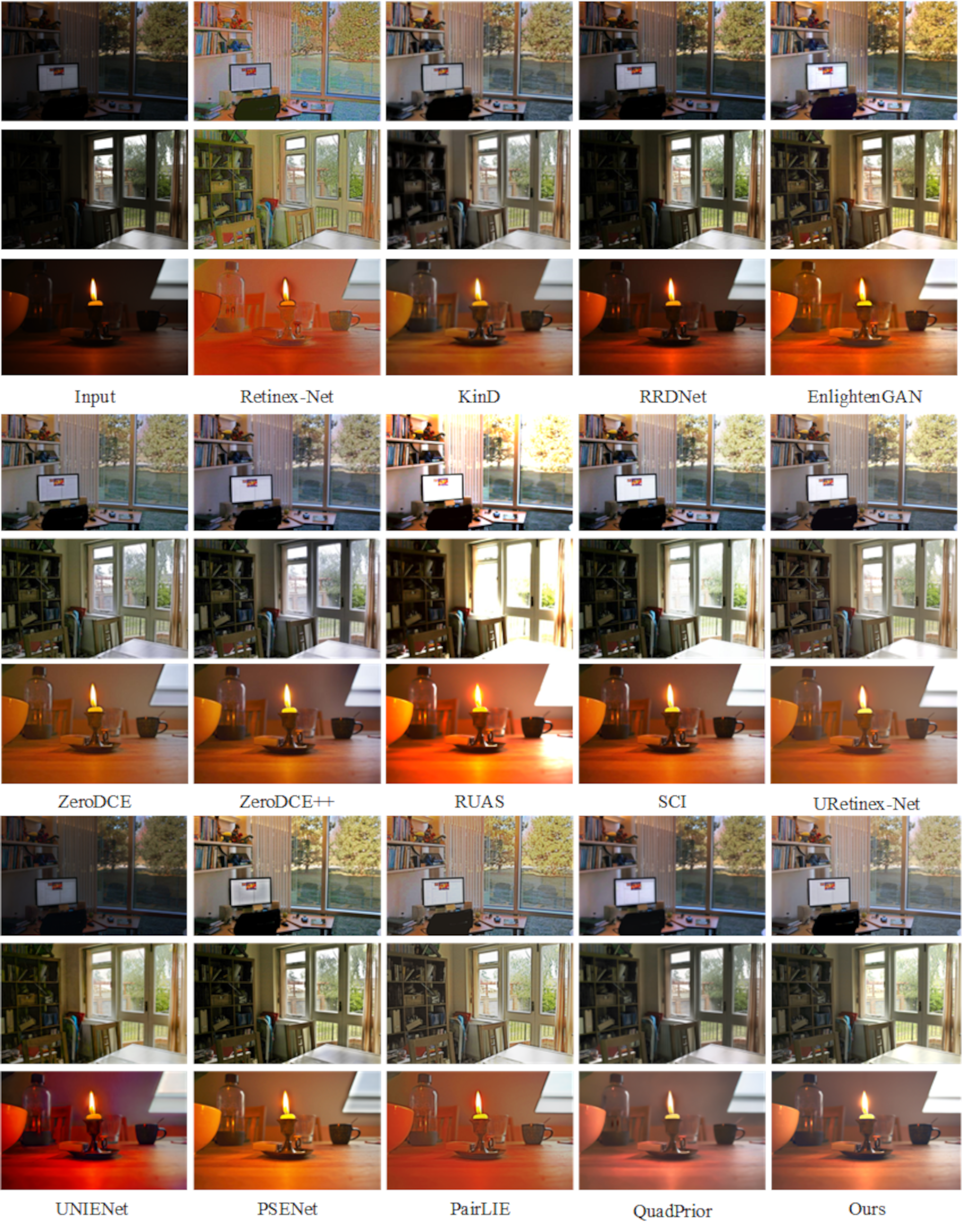

Supplement: S1 File — (ZIP) [file pone.0314541.s001.zip › Supporting Information/Fig 13.tif]

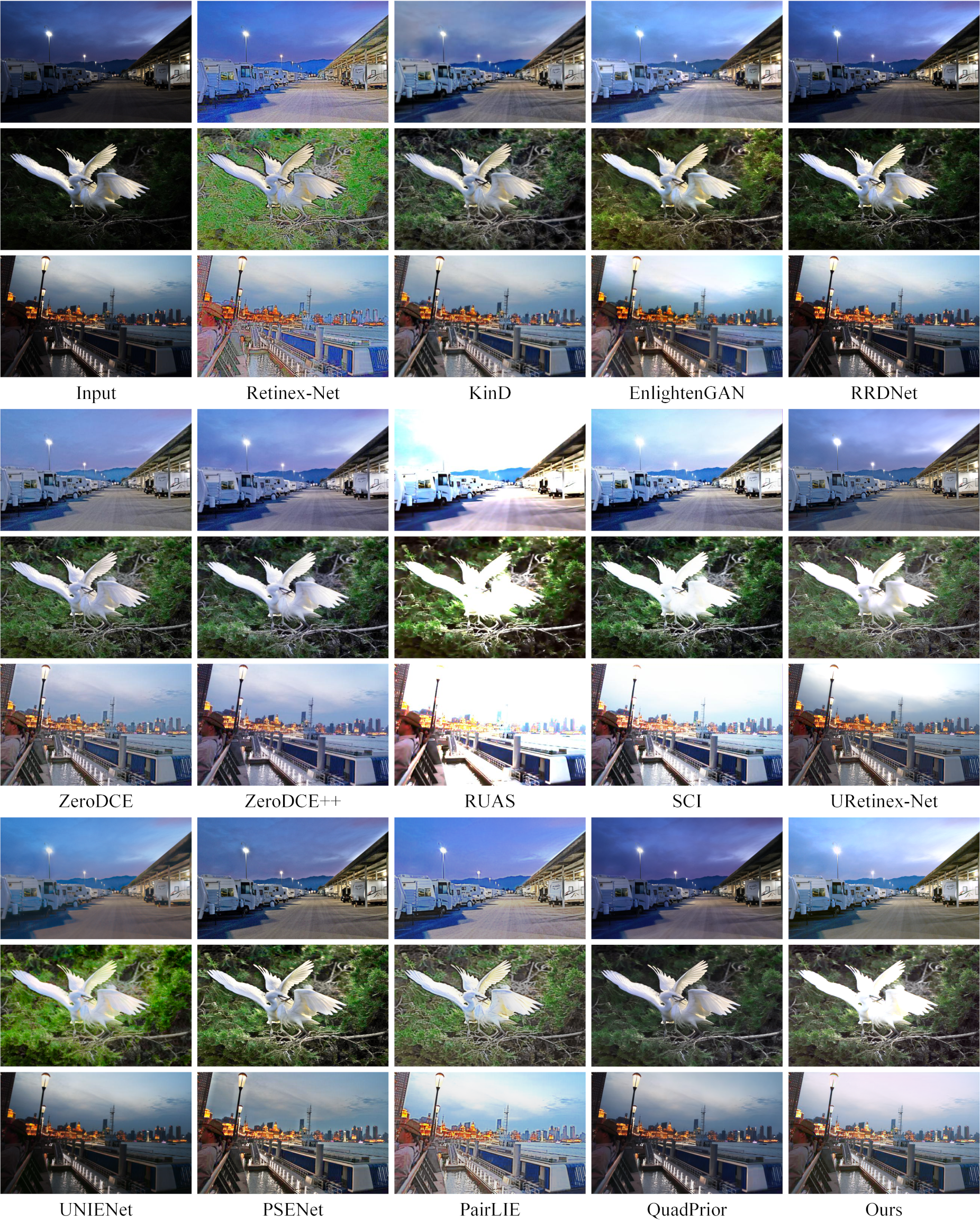

Supplement: S1 File — (ZIP) [file pone.0314541.s001.zip › Supporting Information/Fig 14.tif]

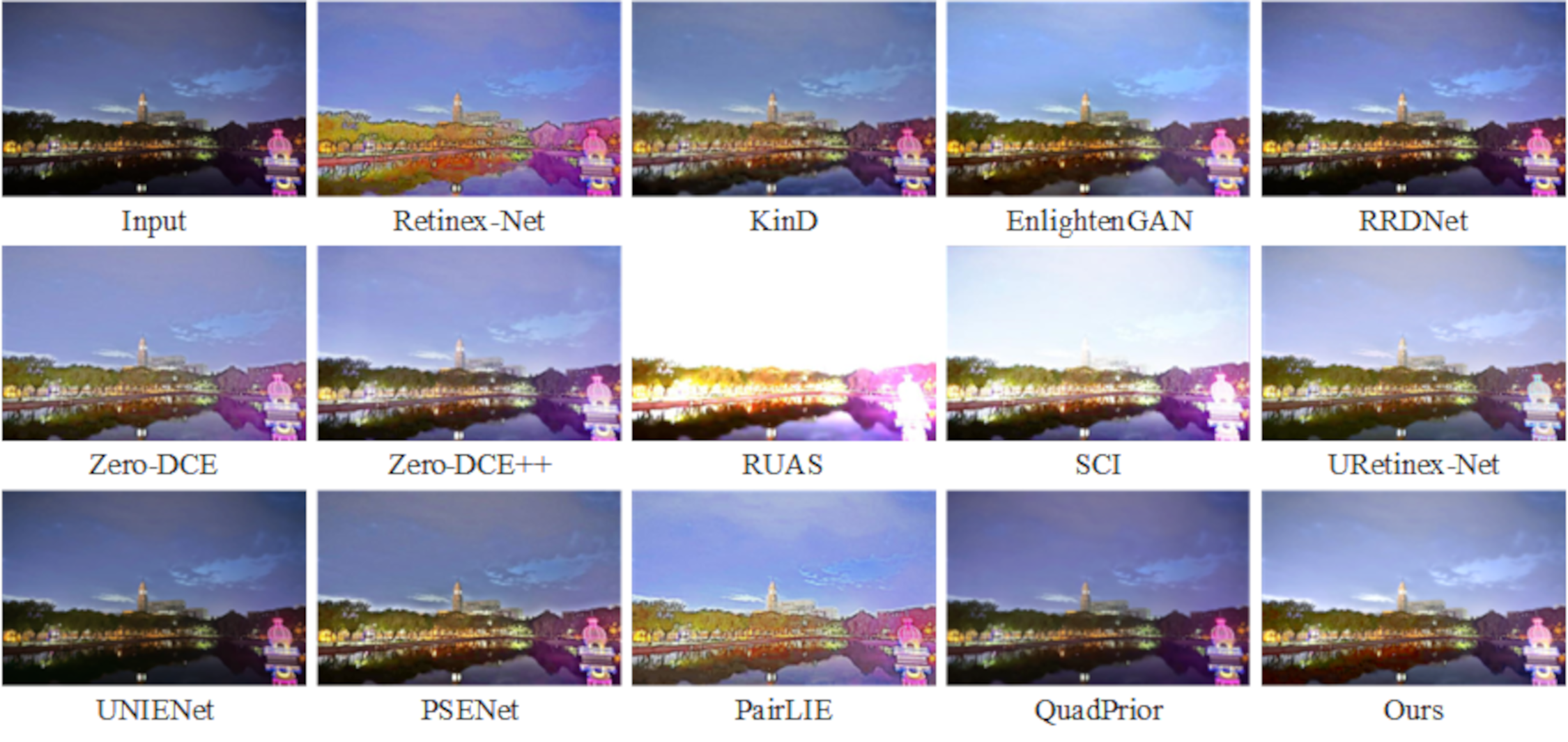

Supplement: S1 File — (ZIP) [file pone.0314541.s001.zip › Supporting Information/Fig 15.tif]

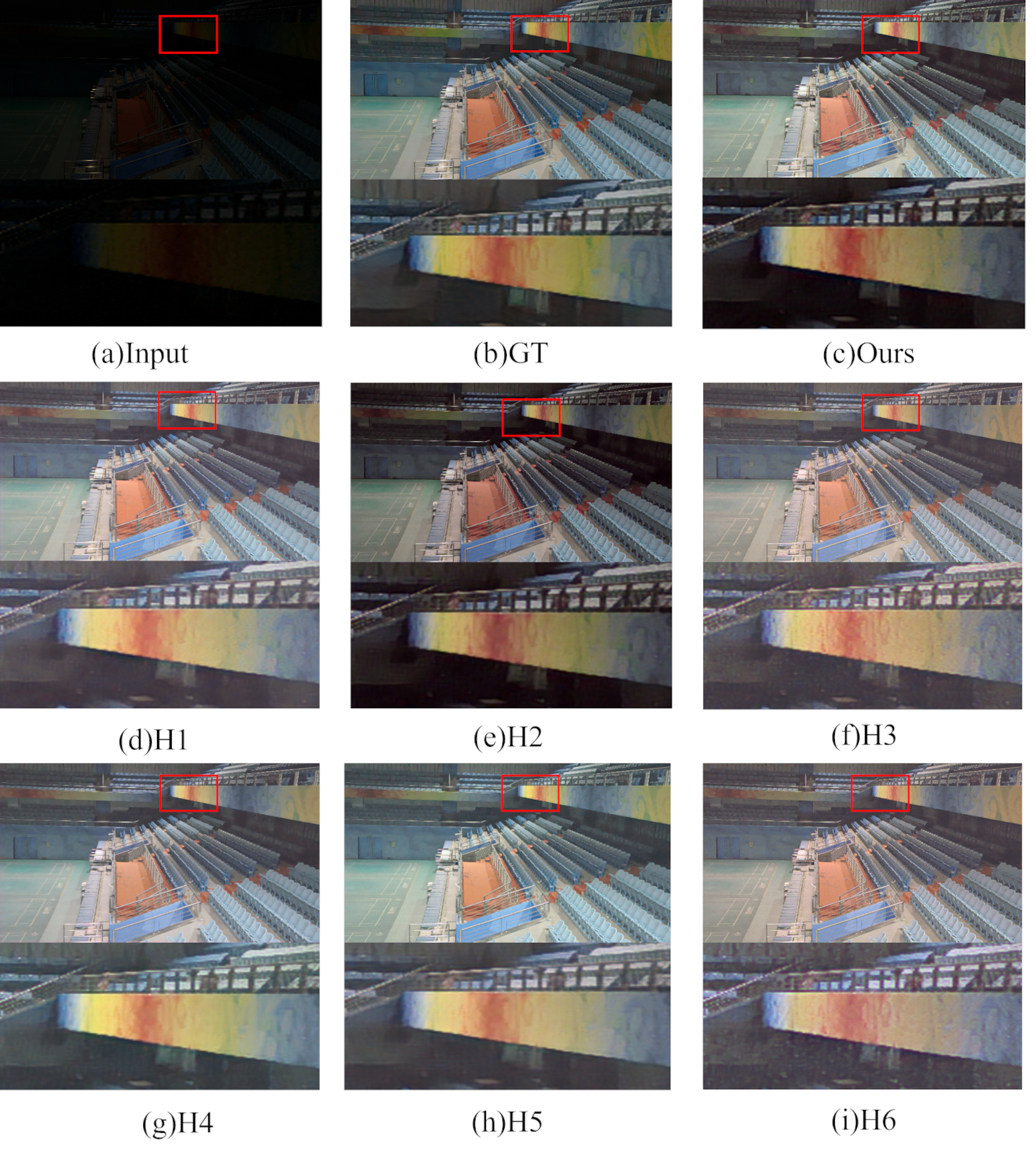

Supplement: S1 File — (ZIP) [file pone.0314541.s001.zip › Supporting Information/Fig 16.tif]

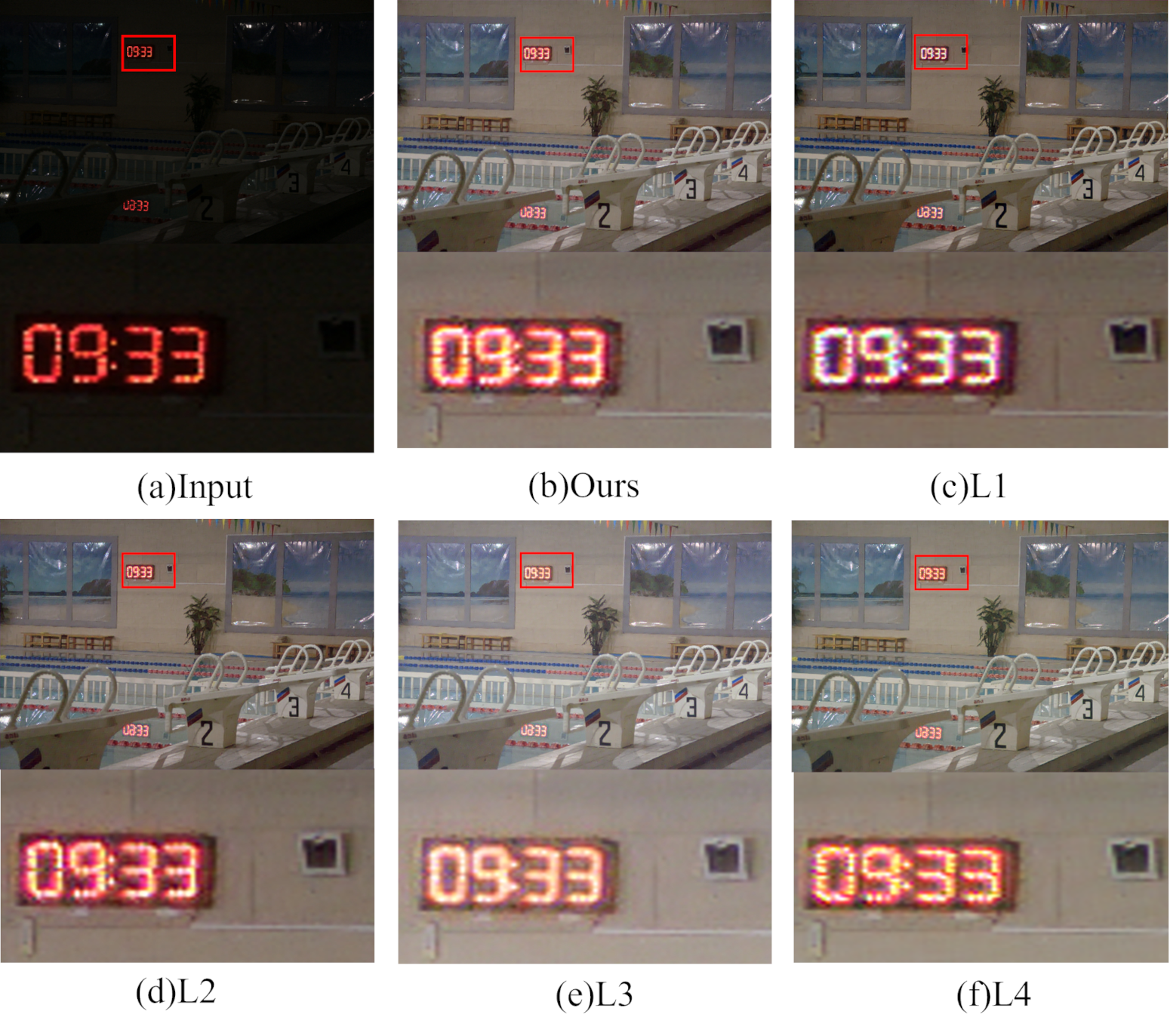

Supplement: S1 File — (ZIP) [file pone.0314541.s001.zip › Supporting Information/Fig 17.tif]

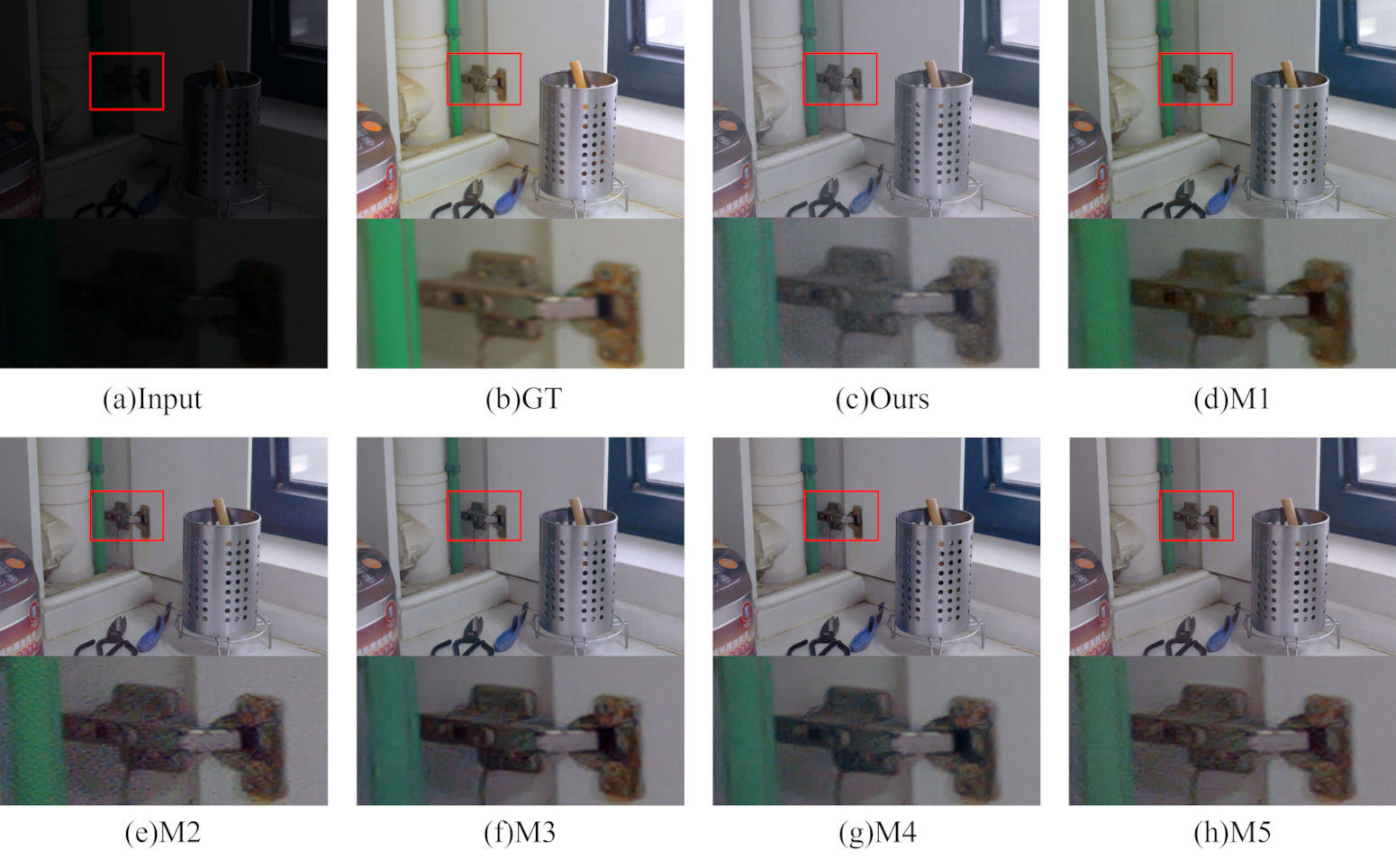

Supplement: S1 File — (ZIP) [file pone.0314541.s001.zip › Supporting Information/Fig 18.tif]

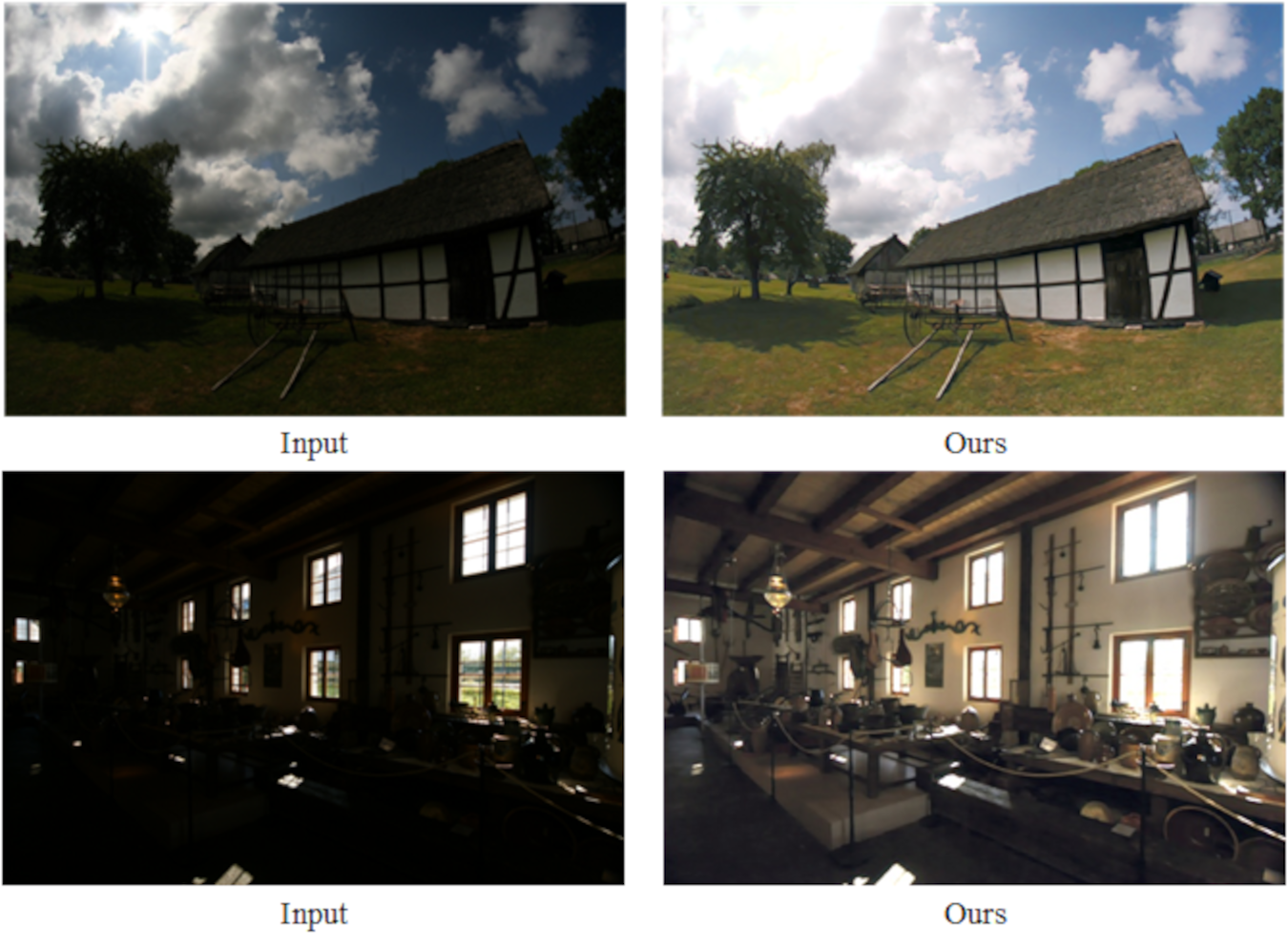

Supplement: S1 File — (ZIP) [file pone.0314541.s001.zip › Supporting Information/Fig 19.tif]

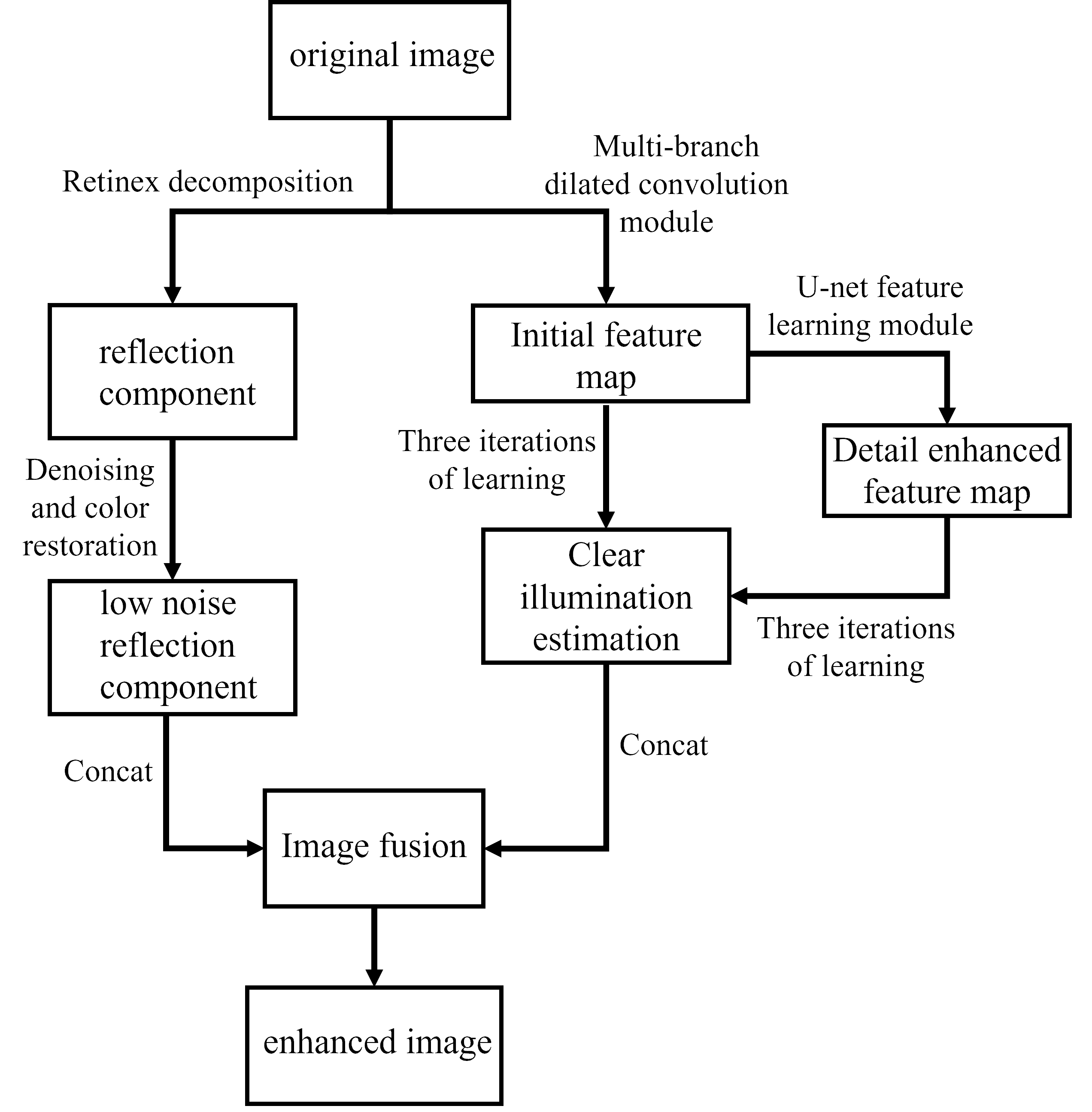

Supplement: S1 File — (ZIP) [file pone.0314541.s001.zip › Supporting Information/Fig 2.tif]

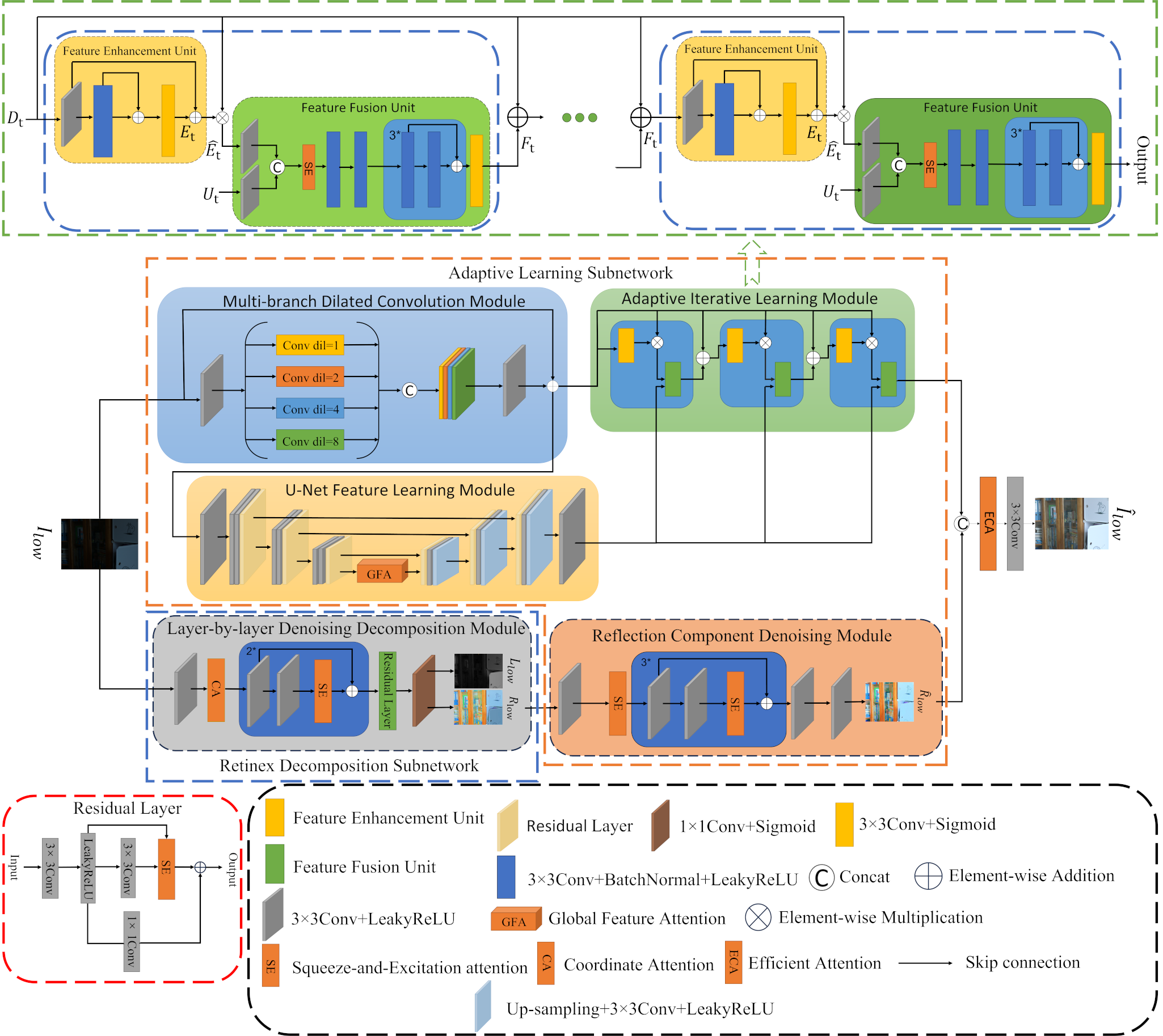

Supplement: S1 File — (ZIP) [file pone.0314541.s001.zip › Supporting Information/Fig 3.tif]

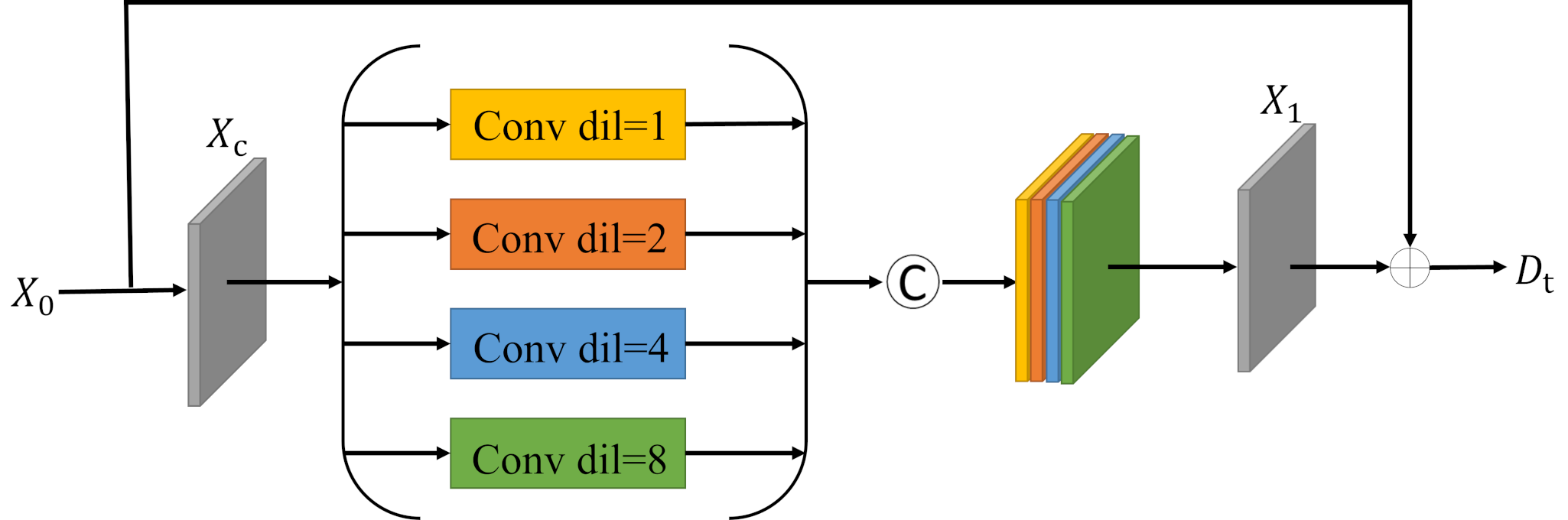

Supplement: S1 File — (ZIP) [file pone.0314541.s001.zip › Supporting Information/Fig 4.tif]

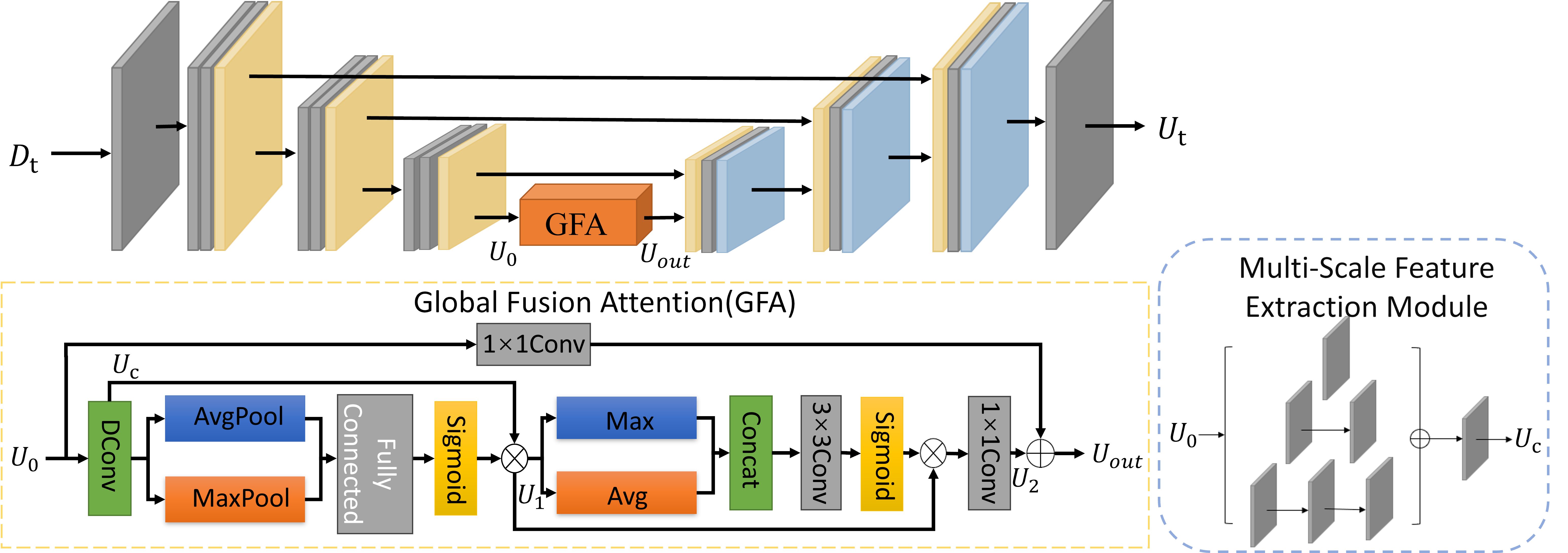

Supplement: S1 File — (ZIP) [file pone.0314541.s001.zip › Supporting Information/Fig 5.tif]

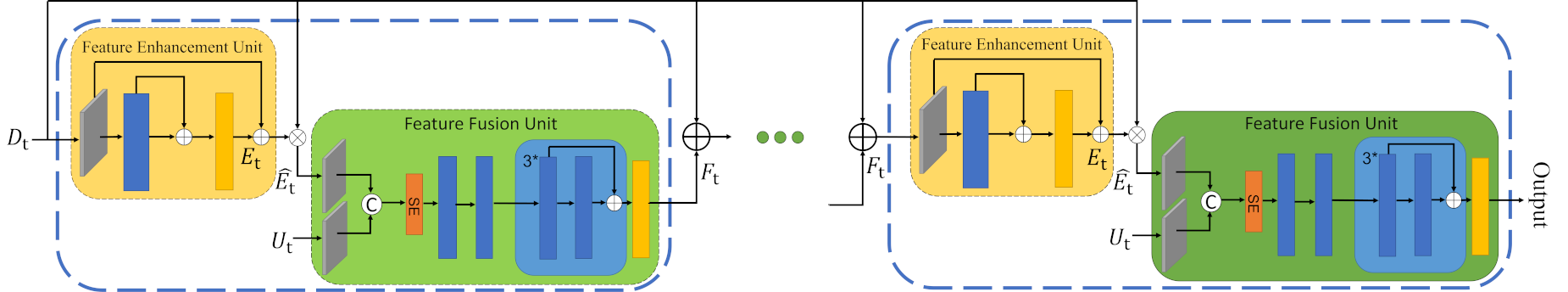

Supplement: S1 File — (ZIP) [file pone.0314541.s001.zip › Supporting Information/Fig 6.tif]

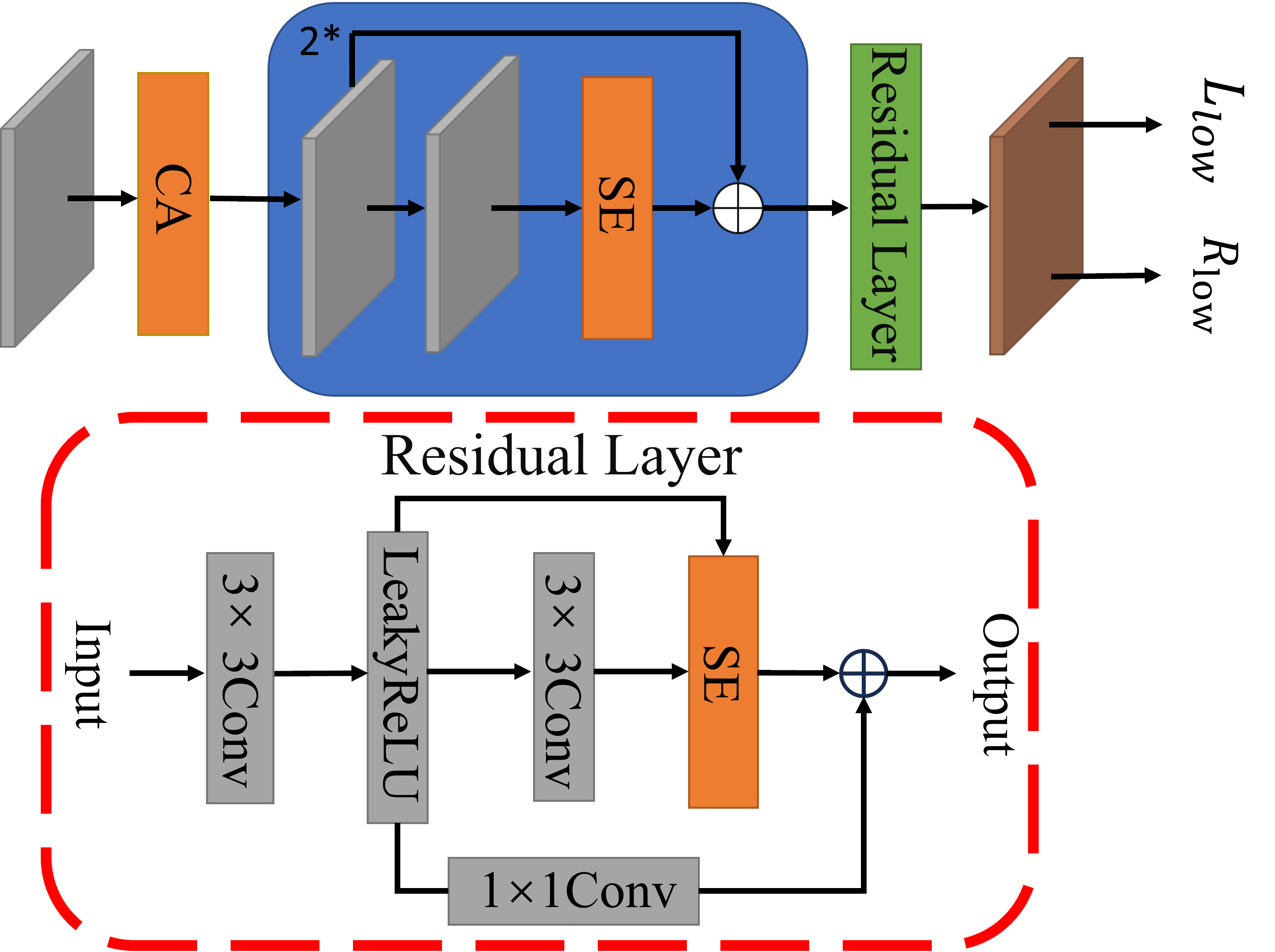

Supplement: S1 File — (ZIP) [file pone.0314541.s001.zip › Supporting Information/Fig 7.tif]

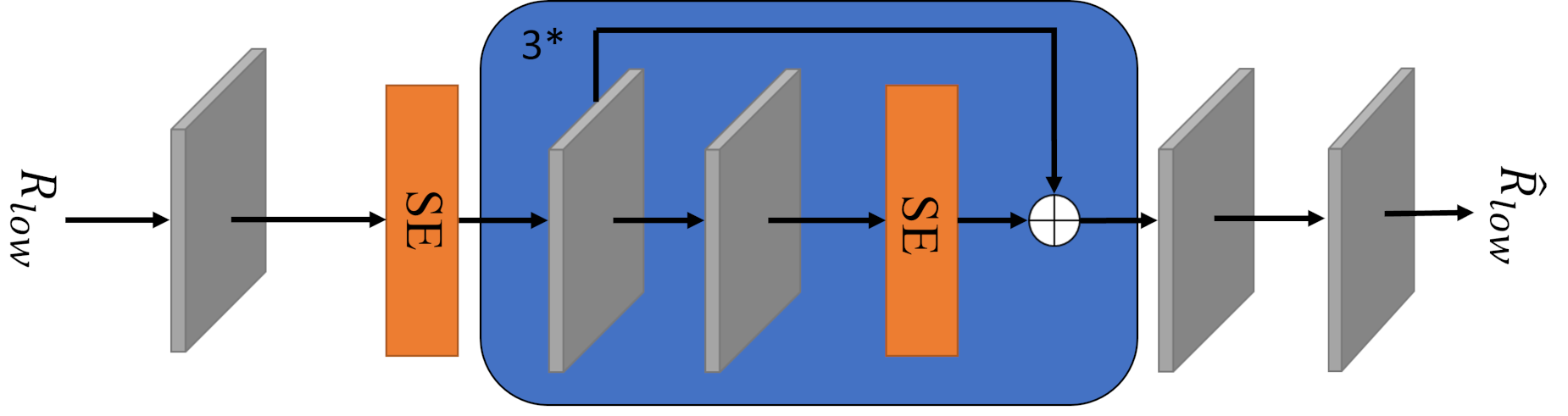

Supplement: S1 File — (ZIP) [file pone.0314541.s001.zip › Supporting Information/Fig 8.tif]

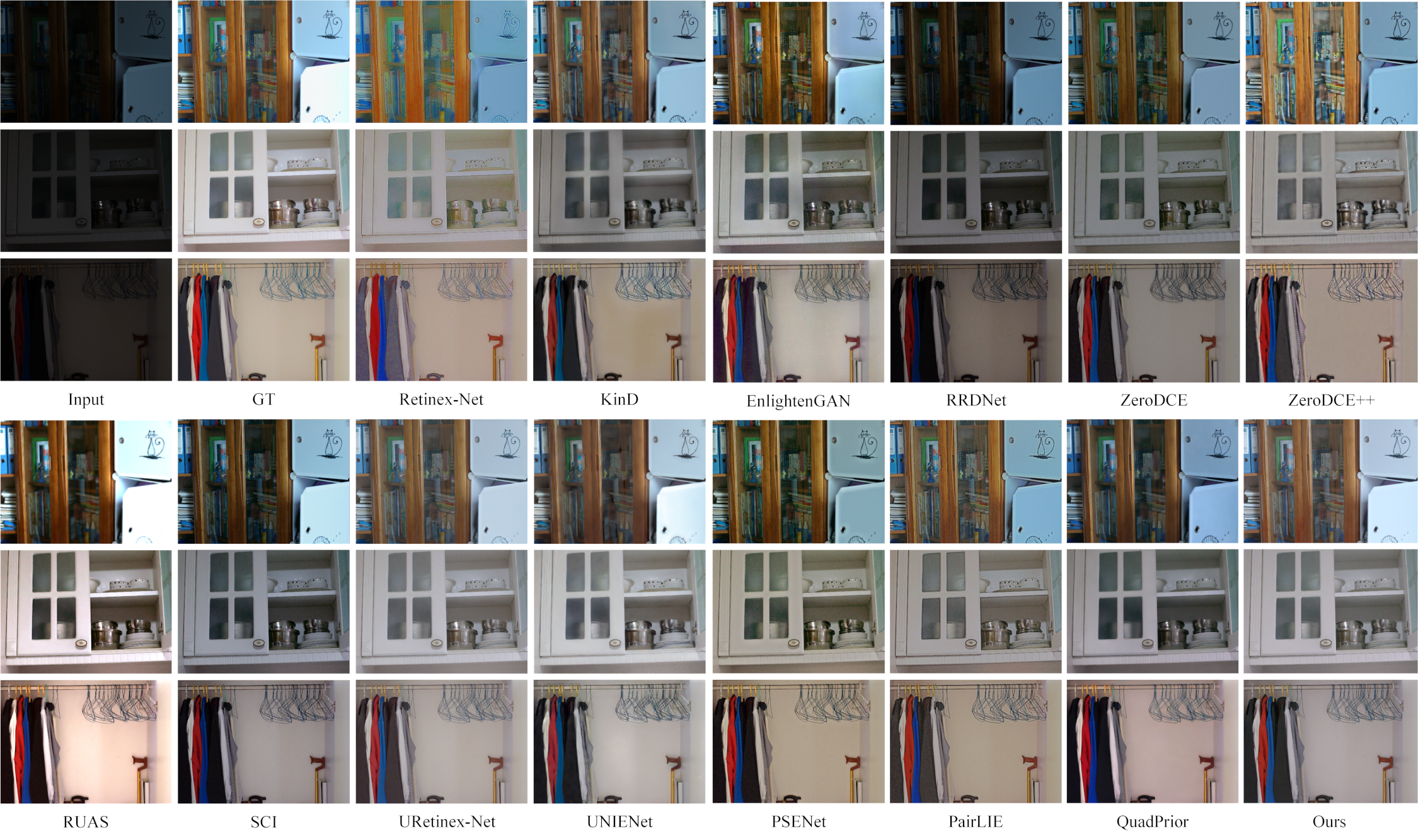

Supplement: S1 File — (ZIP) [file pone.0314541.s001.zip › Supporting Information/Fig 9.tif]
